# Phenolic-enriched ethanol-soluble fraction of *Codonopsis pilosula* attenuates LPS-induced acute lung injury in mice via the gut-lung axis

**DOI:** 10.3389/fphar.2026.1863500

**Published:** 2026-10-07

**Authors:** Jiao Li, Qian Xiao, Yi Ran Sun, Si Yu Zhang, Xun Gou, Jie Xie, Ying Li, Jia Hui Yuan, Ling Ling Zeng, Guang Bin Meng, Jian Xin Chai, Xiao Yu Zhang

**Affiliations:** 1 College of Life Sciences, Sichuan Normal University, Chengdu, China; 2 School of Pharmacy, Chengdu Medical College, Chengdu, China; 3 Shanxi Zhendong Wuhe Yi Yang Tang Co., Ltd., Changzhi, China; 4 Shanxi Key Laboratory of Medicinal and Edible Homology Functional Food, Changzhi, China

**Keywords:** *Codonopsis pilosula* phenolic preparation (CPPP), gut microbiota, gut-lung axis, intestinal barrier, LPS-induced acute lung injury

## Abstract

**Background:**

*Codonopsis pilosula* (Dangshen), a traditional Chinese medicine targeting the Lung Meridian, is commonly steeped in 50% (alcohol by volume) distilled spirits (Baijiu) in China as a medicinal liquor for protecting against lung diseases, including acute lung injury (ALI). This alcohol-steeping preparation yields an ethanol-soluble fraction with a distinct phytochemical profile from conventional aqueous extracts. However, its bioactive compounds and the underlying protective mechanisms against ALI remain unclear.

**Objective:**

This study aimed to characterize the bioactive compounds of the ethanol-soluble fraction of C. *pilosula* (designated as CPPP) and investigate its protective mechanisms against lipopolysaccharide (LPS)-induced ALI in mice.

**Methods:**

CPPP was characterized by non-targeted metabolomic profiling and targeted quantitative analysis using UPLC-ESI-MS/MS, performed sequentially to achieve comprehensive metabolite coverage and precise quantification of identified constituents. Male C57BL/6J mice were pretreated with CPPP for 30 days prior to intraperitoneal injection of LPS to induce ALI. Comprehensive assessments included 16S rRNA sequencing of the gut microbiota, untargeted metabolomic profiling of feces and serum, enzyme-linked immunosorbent assay (ELISA) quantification of serum inflammatory factors and LPS, histological evaluation and immunohistochemistry (IHC) analysis of gut and lung tissues.

**Results:**

CPPP was enriched in phenolic acids and derivatives (25.3%, w/w). Compared to the normal control, CPPP significantly (P < 0.05) enriched beneficial gut bacteria *Clostridium, Bifidobacterium* and *Akkermansia*. CPPP increased fecal levels of butyric acid, serotonin and spermidine, elevated serum level of serotonin, and reduced serum levels of trimethylamine N-oxide, phenylacetylglycine, and phenylacetylglutamine. CPPP significantly (P < 0.001) decreased the levels of LPS-induced pro-inflammatory factors in serum (CRP, TNF-α, IL-6) and lung tissue (MPO, TNF-α, IL-1β, IL-6, IL-18), while increasing the level of anti-inflammatory IL-10 in serum (P < 0.001) and lung tissue (P < 0.01). These anti-inflammatory effects of CPPP, together with the microbiota-derived metabolic alterations, correlated strongly with the CPPP-mediated preservation of intestinal barrier parameters (villus height, goblet cell density, and tight junction protein expression) and attenuation of lung injury parameters (pulmonary inflammation, edema, and histopathological damage).

**Conclusion:**

CPPP attenuated LPS-induced ALI in mice by remodeling gut microbiota, reprogramming key metabolites and suppressing pro-inflammatory factors along the gut-lung axis. These findings validate the lung-protective potential of phenolic compounds from traditional alcohol-steeping preparation of C. *pilosula* and provide mechanistic insights for developing microbiota-targeted interventions in inflammatory lung diseases.

## 1 Introduction

*Codonopsis pilosula* (Franch.) Nannf. (Chinese name: Dangshen), a perennial herbaceous species of the Campanulaceae family, is a traditional Chinese medicine commonly recognized for its effects of augmenting qi, strengthening the spleen, and benefiting the lung. According to Traditional Chinese Medicine theory, *Codonopsis pilosula* is associated with the Lung Meridian (China N.P.C.o.t.P.s.R.o, 2020). Modern pharmacological studies have investigated its potential in inflammatory lung diseases, characterized by exaggerated pulmonary immune responses, such as chronic obstructive pulmonary disease and acute lung injury (ALI) (Bao et al., 2026; Gong et al., 2022). These therapeutic effects are primarily attributed to its bioactive constituents, among which *C. pilosula* polysaccharides derived from aqueous extracts are considered as the principal active components (Lu et al., 2023).

In China, *C. pilosula* is widely incorporated into daily dietary practices, including tea, liquor, soup, and porridge. Among these preparations, *C. pilosula* Liquor (Chinese name: Dangshen Lujiu), a traditional healthcare alcoholic beverage produced by steeping dried *C. pilosula* roots in approximately 50% (alcohol by volume, ABV) distilled spirits (Baijiu), holds particular significance. This alcohol-based extraction utilizes the solubilizing capacity of the hydroalcoholic matrix to selectively enrich lipophilic bioactive compounds, which are poorly soluble in conventional aqueous preparations, while high-molecular-weight polysaccharides are precipitated and thus absent from the final product. Consequently, *C. pilosula* Liquor yields an ethanol-soluble fraction that is expected to possess a phytochemical profile distinct from conventional aqueous preparations. However, its chemical composition and biological activities, particularly its potential effects on inflammatory lung diseases, remain to be characterized.

Existing research on inflammatory lung diseases has revealed an involvement of the gut-lung axis (Liu et al., 2026). This bidirectional communication pathway connects gut microbiota to lung immunity via multiple mechanisms, including microbial metabolites, immune cell infiltration, and systemic inflammatory signaling (Wang L. et al., 2023). A balanced gut microbiota and its metabolites, particularly SCFAs, enhance intestinal barrier integrity, thereby limiting systemic inflammation and conferring lung protection by inhibiting pro-inflammatory cytokines, inducing anti-inflammatory cytokines, and regulating immune cell infiltration (Verma et al., 2024). Conversely, intestinal barrier dysfunction and microbial dysbiosis are intimately associated with impaired lung defense and increased susceptibility to respiratory infections, highlighting the critical role of gut-derived signaling in preserving lung immune homeostasis against pathological challenges (Ashique et al., 2022; Liu et al., 2026). Given that *C. pilosula* polysaccharides confer liver protective effects through the gut-liver axis (Zou et al., 2023), other fractions of *C. pilosula* might similarly target alternative gut-organ axes. Therefore, we hypothesized that the ethanol-soluble fraction of *C. pilosula*, despite lacking polysaccharides, might alternatively modulate the gut-lung axis to confer comparable lung protective effects. Therefore, this study aimed to investigate whether the ethanol-soluble fraction exerts lung protection through the gut-lung axis, thereby expanding the mechanistic understanding of *C. pilosula* beyond its well-characterized polysaccharide constituents.

Acute lung injury (ALI) is an inflammatory lung disease triggered by bacterial infection, with lipopolysaccharide (LPS) from Gram-negative bacteria serving as a critical risk factor (Mowery et al., 2020). ALI is characterized by diffuse alveolar damage, pulmonary edema, profound pulmonary and systemic inflammation, and its pathogenesis is closely associated with dysregulation of the gut-lung axis (Tang et al., 2021; Yi et al., 2026). To test the hypothesis that modulation of the gut-lung axis confers lung protection, this study employed an LPS-induced mouse model of ALI to evaluate the protective effects of the ethanol-soluble fraction derived from C. *pilosula*. Identification and quantification of its major phytochemical constituents were performed to establish a phytochemical basis for the observed effects. These findings provide a foundation for developing the ethanol-soluble fraction of *C. pilosula* as potential adjunctive therapies for inflammatory lung diseases.

## 2 Materials and methods

### 2.1 Materials and chemicals

*Codonopsis pilosula* (Franch.) Nannf. (cultivar: Lu Dangshen) used in this study was manufactured by Shanxi Zhendong Wuhe Yi Yang Tang Co., Ltd. (Shanxi, China) and conformed to the quality standards of the Chinese Pharmacopoeia (2020 edition) (China N.P.C.o.t.P.s.R.o, 2020). Authentication was performed by Prof. Xiao Yu Zhang from College of Life Sciences, Sichuan Normal University using a combination of macroscopic and microscopic methods, in accordance with the identification tests specified for *Codonopsis pilosula* in the Chinese Pharmacopoeia (2020 edition). Macroscopically, the dried root slices exhibited the characteristic yellowish-brown to greyish-brown outer surface, a firm yet slightly pliable texture, and a distinct, sweet-tasting, aromatic fracture. Microscopically, powdered samples were cleared with chloral hydrate and examined under a light microscope. Key characteristic features consistently observed included abundant inulin crystal masses, scattered stone cells with moderately thickened and pitted walls, fragments of bordered-pitted vessels, and broken laticifer segments containing yellowish-brown granular contents. To further confirm botanical identity and quality, the ethanol-soluble fraction of the authenticated material was analyzed by UPLC-ESI-MS/MS. The characteristic bioactive markers lobetyolin, syringin and tangshenoside I were detected and quantified, with lobetyolin serving as the primary pharmacopoeial marker and the latter two as essential quality control constituents. Voucher specimens of the decoction pieces used in the study were deposited at the same institution under accession number SNU-LS-CP-LD-2024-021.

LPS from *Escherichia coli* O1111:B4 was purchased from Sigma-Aldrich (UNITED STATES). The 12 external reference standards were purchased from Chengdu Bencao Tianyun Biotechnology Co., Ltd. (Chengdu, China): Lobetyolin (Chemical Abstracts Service Number: 136085-37-5), Tangshenoside I (117278-74-7), Syringin (118-34-3), Atractylenolide III (73030-71-4), Neochlorogenic acid (906-33-2), Chlorogenic acid (327-97-9), Cryptochlorogenic acid (905-99-7), Caffeic acid (331-39-5), Ferulic acid (1135-24-6), Cinnamic acid (140-10-3), p-Coumaric acid (501-98-4), Quinic acid (77-95-2). Chromatographic-grade solvents (acetonitrile, methanol, formic acid and ammonium formate) and analytical-grade reagents were obtained from Chengdu Shuobo Yanchuang Science & Technology Co., Ltd. (Chengdu, China).

### 2.2 Preparation of the powder of ethanol-soluble fraction of *C. pilosula*

The samples were prepared according to the traditional processing method of *C. pilosula* Liquor. 500 g of dried C. *pilosula* root slices were subjected to static maceration in 4,000 mL of 50% (alcohol by volume, ABV) distilled spirit (material-to-liquid ratio of 1:8, w/v) for 60 days without agitation. After filtration, the resulting extract was concentrated under reduced pressure and subsequently freeze-dried to obtain the powder of ethanol-soluble fraction of C. *pilosula*, which was stored at 4 °C for subsequent use.

### 2.3 Non-targeted metabolomic analysis of ethanol-soluble fraction of *C. pilosula*

The sample for analysis was prepared by dissolving 30 mg of the powder of ethanol-soluble fraction of C. *pilosula* in 1.5 mL of ice-cold 70% (v/v) methanol. The mixture was vortexed for 30 s every 30 min, six times. After centrifugation at 12,000 rpm for 3 min, the supernatant was filtered through a 0.22 μm membrane to yield the analytical sample.

Non-targeted analysis was performed using an integrated Ultra Performance Liquid Chromatography-Electrospray Ionization-Tandem Mass Spectrometry (UPLC-ESI-MS/MS) system (ExionLC™ AC coupled with QTRAP 6500 mass spectrometer (SCIEX, UNITED STATES). Chromatographic separation was achieved on an Agilent SB-C18 column (100 mm × 2.1 mm, 1.8 μm). The mobile phase consisted of 0.1% (v/v) formic acid in water (solvent A) and 0.1% (v/v) formic acid in acetonitrile (solvent B). The following gradient elution program was applied at a flow rate of 0.35 mL/min and a column temperature of 40 °C: 0–9 min, 5%–95% B; 9–10 min, 95% B; 10–11.1 min, 95%–5% B; 11.1–14 min, 5% B for equilibration. The injection volume was 2 μL. Mass spectrometric detection was conducted using an ESI source. The source temperature was set to 500 °C. The ion spray voltage (IS) was set at 5500 V in positive mode and −4500 V in negative mode. The ion source gas I (GSI), gas II (GSII), and curtain gas (CUR) were set to 50, 60, and 25 psi, respectively. The collision-activated dissociation (CAD) gas was set to “High”. Analyses were performed using multiple reaction monitoring (MRM) experiments on a triple quadrupole instrument. The collision gas (nitrogen) was set to “Medium”. The declustering potential and collision energy were optimized for each MRM transition. Specific sets of MRM transitions were monitored according to the elution period of the metabolites.

### 2.4 Targeted quantification of major components of ethanol-soluble fraction of *C. pilosula*

Based on combined fingerprint and MS profiling obtained from non-targeted metabolomic analysis of ethanol-soluble fraction of *C. pilosula*, 12 representative compounds corresponding to the characteristic DAD peaks were selected for targeted quantification using external reference standards. This qualitative and quantitative analysis was performed on a UPLC-DAD-MS system, which consisted of an Acquity H-CLASS System (Waters, UNITED STATES) equipped with an ACQUITY Premier HSS T3 Column (100 mm × 2.1 mm, 1.8 μm). The mobile phase consisted of 0.1% (v/v) formic acid and 25 mM ammonium formate in water (solvent A) and acetonitrile (solvent B). The following gradient elution program was applied at a flow rate of 0.5 mL/min and a column temperature of 40 °C: 0∼3 min, 1% B; 3–12.1 min, 1%–20% B; 12.1–18 min, 20%–45% B; 18–24 min, 45% B; 24–25.1 min, 45%–1% B; 25.1–30 min, 1% B for equilibration. The injection volume was 1 µL. Detection was carried out using a diode array detector (DAD) at 280 nm, 300 nm, and 320 nm.

Mass spectrometric detection was conducted using an electrospray ionization (ESI) source in both positive and negative ionization modes. The operating parameters were as follows: cone voltage, 15 V (positive mode) and 30 V (negative mode); capillary voltage, 0.8 kV; probe temperature, 600 °C; source temperature, 126 °C; and desolvation gas flow, 0.25 mL/min. Data were acquired over a mass range of m/z 150–900 with a sampling rate of 10 points per peak.

### 2.5 Animal experiments

#### 2.5.1 Laboratory animals

All animal care and experimental protocols employed in this study were reviewed and approved by the Ethics Committee of Sichuan Normal University (Approval No. 2025LS037). Nine-week-old male C57BL/6J mice (weighing 20–23 g) were purchased from Chengdu Dashuo Laboratory Animal Technology Co., Ltd. (Chengdu, China; SCXK 2025-0030) and acclimatized for 2 weeks under specific-pathogen-free conditions (temperature: 20 °C-22 °C; relative humidity: 40%–60%; 12 h light/dark cycle) with free access to water and experimental diet (with detailed information in Supplementary Tables S1, S2).

#### 2.5.2 Establishment and grouping of animal models of LPS-induced ALI

Mice were randomly assigned to four groups as follows: (1) The normal control group (namely, NC, n = 10): administered daily by oral gavage with 0.9% (w/v) sterile saline. (2) The ethanol solvent control group (namely, EtOH, n = 5): administered daily by oral gavage with a vehicle solution prepared by diluting anhydrous ethanol in 0.9% sterile saline to a final ABV of 50%. Gavage volume was standardized at 10 mL/kg body weight as a 50% (v/v) ethanol solution, delivering a pure ethanol dose of 5 mL/kg (approximately 3.95 g/kg, based on an ethanol density of 0.789 g/mL). (3) The CPPP treatment group (namely, CPPP, n = 5): administered daily by oral gavage with a suspension of the lyophilized CPPP powder in 0.9% sterile saline at a dose of 250 mg/kg body weight, standardized to total active constituents, in a final gavage volume of 10 mL/kg. The lyophilized powder was prepared by complete evaporation of ethanol from the ethanol-soluble fraction of *C. pilosula*, leaving negligible residual ethanol. (4) The ethanol solvent + CPPP group (namely, EtOH + CPPP, n = 5): administered daily by oral gavage with a suspension of the lyophilized CPPP powder (250 mg/kg) prepared in the identical ethanol-saline vehicle as described for the EtOH group. Specifically, CPPP powder was first suspended in 5 mL/kg of 50% (v/v) ethanol solution and then brought to a final volume of 10 mL/kg with 0.9% sterile saline. This ensured that the EtOH + CPPP group received an identical pure ethanol dose (5 mL/kg, approximately 3.95 g/kg), identical final gavage volume (10 mL/kg), and identical final ABV (50%, v/v) as the EtOH group. The gavage volume was standardized to 10 mL/kg body weight across all groups. After a 30-day intervention period of continuous oral gavage, the feces were collected from all mice prior to the first LPS injection and stored at −80 °C for 16S rRNA sequencing.

ALI was induced by two consecutive intraperitoneal injections of LPS. The NC mice group were split at random into two subgroups: the NC/LPS- subgroup (n = 5): received intraperitoneal injection of 0.9% sterile saline; the NC/LPS + subgroup (n = 5) and all treatment groups (EtOH/LPS+, EtOH + CPPP/LPS+, CPPP/LPS+, n = 5 for each group): received a first intraperitoneal injection of LPS (2 mg/kg dissolved in 0.9% sterile saline), followed 24 h later by a second injection of LPS (5 mg/kg dissolved in 0.9% sterile saline). 6 h after the second LPS injection, all mice were weighed and then anesthetized with intraperitoneal injection of sodium pentobarbital (30 mg/kg dissolved in 0.9% sterile saline). Blood was collected by cardiac puncture, allowed to clot at room temperature for 30 min, and centrifuged at 3,000 × g for 15 min at 4 °C. The resulting serum was separated, aliquoted, and stored at −80 °C for subsequent metabolomic analysis of gut microbiota-derived metabolites and for quantification of inflammatory factors and LPS. The lungs and intestines were excised for subsequent histopathological analysis. Detailed experimental procedures are shown in Figure 1A.

**FIGURE 1:**
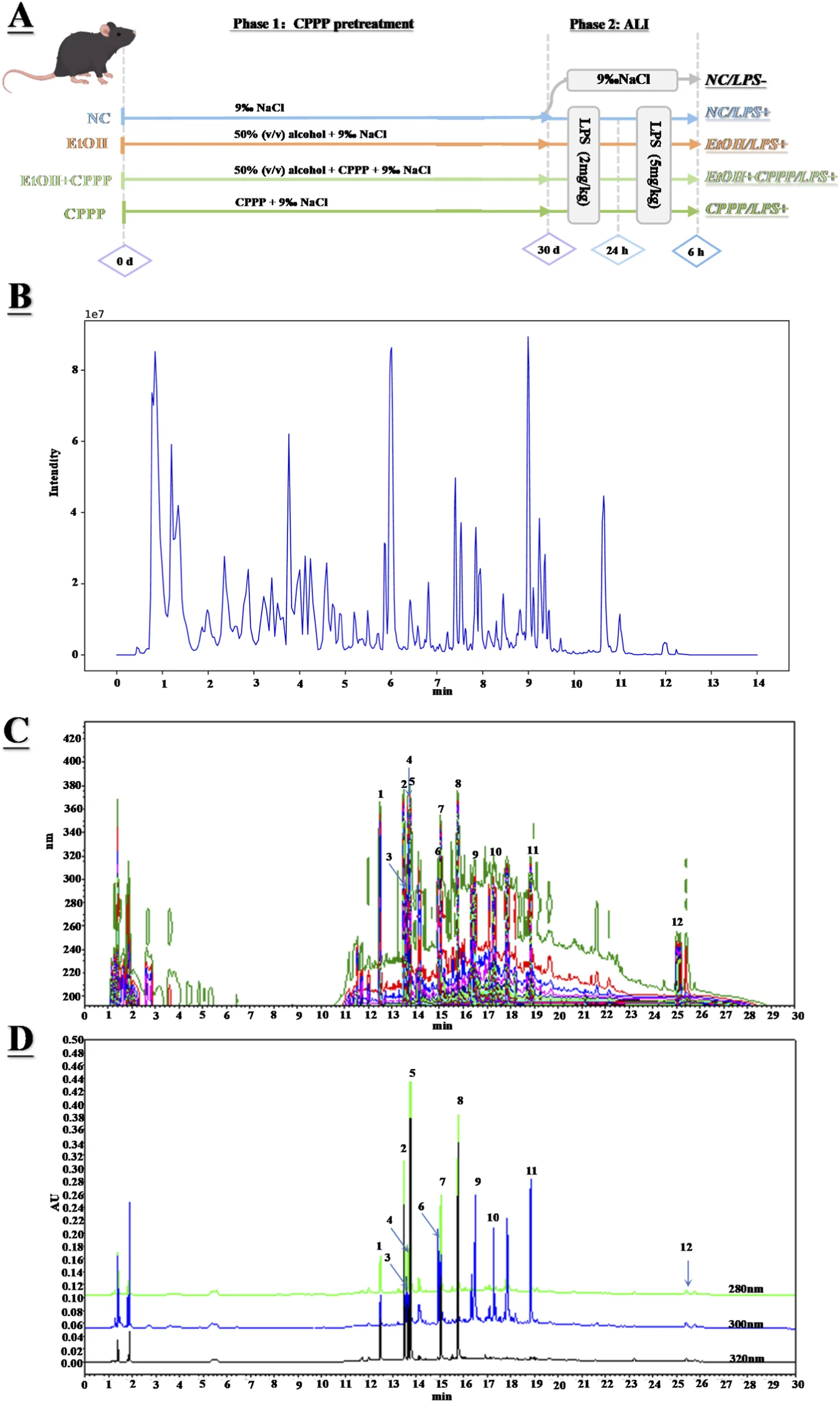
Experimental process **(A)**, total ion chromatogram of CPPP obtained from non-targeted MS analysis **(B)**, UPLC-DAD chromatogram of CPPP at multiple wavelengths **(C)** and UPLC overlaid chromatogram of CPPP at 280, 300 and 320 nm wavelengths **(D)**.

### 2.6 Calculation of the wet weight-to-dry weight (W/D) ratio of lung tissues

The right middle lobe of the lung was excised, briefly blotted on absorbent paper to remove surface fluid, and immediately weighed to obtain its wet weight (W). It was then placed in an oven at 70 °C for 72 h until a constant weight was achieved, at which point its dry weight (D) was measured. The extent of pulmonary edema was assessed by calculating the wet-to-dry weight ratio (W/D).

### 2.7 Histopathological evaluation of the lung and jejunal tissues

The left lung lobes and jejunal tissues were fixed in 10% neutral-buffered formalin for 24–48 h at room temperature, dehydrated through a graded ethanol series (70%, 80%, 95%, and 100%, each for 1 h), cleared in xylene (two times, each for 30 min), and embedded in paraffin. Tissue sections (4–6 μm thickness) were cut using a rotary microtome, mounted on poly-L-lysine-coated glass slides, and dried at 60 °C for 1 h. Sections were deparaffinized in xylene (two times, each for 10 min), rehydrated through descending ethanol concentrations (100%, 95%, 80%, 70%, each for 5 min), and stained with hematoxylin and eosin (HE) staining kit (Beyotime Biotechnology Co., China), following the manufacturer’s instruction. Microscopic examination was performed using a Leica DM2500 microscope (Leica Microsystems, Germany) to assess tissue architecture.

For lung sections, ALI was evaluated using the Mikawa semiquantitative scoring system (Mikawa et al., 2003). Four pathological features were assessed in each section under a light microscope at ×200 magnification by two independent, blinded pathologists: (1) alveolar congestion, (2) hemorrhage, (3) neutrophil infiltration or aggregation in the alveolar space or interstitium, and (4) alveolar septal thickening and/or hyaline membrane formation. Each parameter was graded on a five-point scale as follows: 0 = no injury; 1 = slight injury (<25% of the field); 2 = moderate injury (25–50% of the field); 3 = severe injury (50–75% of the field); and 4 = very severe injury (>75% of the field). The total lung injury score was calculated as the sum of the four individual parameter scores, yielding a value ranging from 0 (no injury) to 16 (maximal injury). The mean score of the two observers was used for statistical analysis.

For jejunal sections, villus height and crypt depth were measured by two independent, blinded pathologists using ImageJ software (version 1.53a, United States); at least 10 well-oriented villi per tissue section were analyzed, and the mean villus height-to-crypt depth ratio was calculated.

For mucus-secreting goblet cell quantification in jejunal sections, Alcian blue-periodic acid-Schiff (AB-PAS) staining was performed. Sections were deparaffinized and rehydrated as described above, stained with AB-PAS staining kit (Beyotime Biotechnology Co., China), following the manufacturer’s instruction. After counterstaining with hematoxylin for 2 min and dehydration, slides were mounted. AB-PAS-positive goblet cells (appearing purple to dark blue) were counted in 10 consecutive, well-oriented crypt-villus units per tissue section at ×200 magnification by two independent, blinded pathologists. The data were expressed as the mean number of goblet cells per crypt-villus unit.

### 2.8 Immunohistochemistry (IHC) analysis of the lung and jejunal tissues

The lung and jejunal tissues were embedded in paraffin and sectioned at 4 μm. The lung tissue sections were incubated with specific primary antibodies against myeloperoxidase (MPO), interleukin-1β (IL-1β), interleukin-18 (IL-18), interleukin-10 (IL-10), interleukin-6 (IL-6), and tumor necrosis factor-α (TNF-α); while the jejunal tissue sections were incubated with primary antibodies targeting the tight junction proteins Occludin, zonula occludens-1 (ZO-1), and Claudin-1. Following primary antibody incubation, appropriate secondary antibodies and detection systems were applied according to standard protocols. Finally, the sections were incubated with a horseradish peroxidase (HRP)-labeled goat anti-rabbit secondary antibody for 2 h at room temperature. After 3,3′-diaminobenzidine (DAB) staining, the sections were imaged using a trinocular digital microscope (BA210Digital, Motic, Xiamen, China). Image analysis was performed using ImageJ2 v2.15.0 (United States).

### 2.9 Measurement of the serum levels of C-reactive protein (CRP), tumor necrosis factor-α (TNF-α), interleukin-6 (IL-6), and interleukin-10 (IL-10) by enzyme-linked immunosorbent assay (ELISA)

The serum was diluted at 1:2 in sample diluent immediately before assay. Serum levels of CRP, TNF-α, IL-6, IL-10 and LPS were measured using the respective MLBIO ELISA kits (Shanghai Enzyme-linked Biotechnology Co., Ltd., Shanghai, China) according to the manufacturer’s instructions. 100 μL of the diluted sample or standard was added per well, and the plate was incubated for 1.5 h at room temperature. Each well was then washed three times with 300 μL of 1× wash buffer. Subsequently, 100 μL of antibody solution was added per well, and the plate was incubated for 1 h at room temperature. After washing, 100 μL of 1× streptavidin-HRP solution was added per well, and the plate was incubated for 30 min at room temperature in the dark. After washing, 50 μL of chromogenic substrate solution A and 50 μL of solution B were added sequentially to each well, and the plate was incubated for 15 min at room temperature in the dark. The reaction was stopped by adding 50 μL of stop solution, and the absorbance was immediately read at 450 nm using a microplate reader (SpectraMax i3x, Molecular Devices, United States). Concentrations were calculated by interpolating from standard curves fitted with a four-parameter logistic equation.

### 2.10 16S rRNA sequencing of the fecal microbiota

Full-length 16S rRNA gene sequencing of murine fecal microbiota was performed on the PacBio Sequel IIe platform (Magigene Bioinformatics Technology, China). Genomic DNA extracted from fecal samples using the FastDNA Spin Kit underwent amplification of the V1-V9 hypervariable regions using the universal bacterial primers 27F (5′-AGRGTTYGATYMTGGCTCAG-3′) and 1492R (5′-RGYTACCTTG TTACGACTT-3′), followed by SMRTbell library construction and circular consensus sequencing (CCS > Q30). Amplicons were sequenced on the Illumina MiSeq platform (Illumina, San Diego, United States) using 2 × 300 bp paired-end chemistry according to the standard protocols of Majorbio Bio-Pharm Technology Co. Ltd. (Shanghai, China). Raw sequencing data were processed in Quantitative Insights Into Microbial Ecology 2 (QIIME2, v2023.2) (Bolyen et al., 2019). Primers were removed, and reads were quality-filtered, denoised, and assembled into amplicon sequence variants (ASVs) at 100% sequence identity using DADA2. Taxonomy was assigned to ASVs against the SILVA v138.1 reference database using a pre-trained Naive Bayes classifier.

For diversity analyses, the ASV table was rarefied to 50,000 reads per sample to normalize sequencing depth. Alpha diversity indices, including Shannon diversity, Chao1 richness, and the number of observed ASVs, were calculated in QIIME2. To compare alpha diversity across groups, the non-parametric Kruskal-Wallis rank-sum test was applied for overall group differences, and pairwise comparisons were performed using the Wilcoxon rank-sum test with Benjamini-Hochberg correction for multiple testing. Beta diversity was evaluated by computing Bray-Curtis dissimilarities based on the ASV abundance table. The resulting distance matrix was visualized using both non-metric multidimensional scaling (NMDS) and principal component analysis (PCA) to assess the overall structural variation of the gut microbial communities among experimental groups. Permutational multivariate analysis of variance (PERMANOVA, 999 permutations) was used to statistically assess differences in overall microbial community structure among groups. Linear discriminant analysis effect size (LEfSe) was performed to identify bacterial taxa significantly enriched in each group, with an LDA score threshold of > 3.0 and a significance level of P < 0.05.

### 2.11 Fecal and serum metabolomic analysis

Aliquots of 50 mg of feces or 50 μL of serum were vortex-mixed with 250 μL of ice-cold 70% (v/v) methanol for 3 min. After centrifugation (12,000 rpm, 10 min, 4 °C), the supernatants were deproteinized and derivatized with 10 mM 3-nitrophenylhydrazine (3-NPH) and 6 mM 1-ethyl-3-(3-dimethylaminopropyl) carbodiimide (EDC) at 40 °C for 60 min. Derivatized samples were analyzed on a QTRAP 6500+ mass spectrometer (SCIEX, United States) coupled to an ExionLC AD ultra-high-performance liquid chromatography (UHPLC) system. Chromatographic separation was performed on an ACQUITY UPLC BEH C18 column (2.1 mm × 100 mm, 1.7 μm; Waters, United States) maintained at 40 °C. The mobile phase consisted of 0.1% formic acid in water (solvent A) and 0.1% formic acid in acetonitrile (solvent B), with a gradient elution program: 0–2 min, 5% B; 2–15 min, 5%–95% B; 15–17 min, 95% B; 17–17.1 min, 95%–5% B; 17.1–20 min, 5% B. The flow rate was 0.4 mL/min, and the injection volume was 2 μL.

Mass spectrometry was operated in MRM mode with ESI source. Source parameters were optimized as follows: ion spray voltage, 5500 V (positive mode) and −4500 V (negative mode); curtain gas, 35 psi; nebulizer gas (GS1), 55 psi; heater gas (GS2), 55 psi; source temperature, 550 °C. Declustering potentials and collision energies were optimized for each MRM transition, and transitions were monitored in scheduled MRM mode with a detection window of 60 s and target scan time of 0.5 s. Data acquisition was controlled by Analyst software (version 1.6.3, SCIEX, United States), and quantification was performed with MultiQuant software (version 3.0.3, SCIEX, United States) using internal standard calibration curves. Subsequent metabolomics data processing, including peak extraction, alignment and normalization, was conducted on the Metware Cloud platform (https://cloud.metware.cn).

### 2.12 Correlation analyses

Pairwise Pearson correlation coefficients were calculated to assess associations between: (1) serum metabolite levels and the relative abundances of gut microbial species; (2) fecal metabolite levels and intestinal barrier markers (villus height, goblet cell numbers, tight junction protein expression); and (3) serum metabolite levels and lung injury indicators (levels of inflammatory factors, pulmonary edema, histopathological scores). All correlations were performed using matched fecal/serum and tissue samples from mice, with P < 0.05 considered statistically significant.

### 2.13 Comparative analysis

Metabolites simultaneously detected in fecal and serum samples were subjected to Pearson correlation analysis against intestinal barrier integrity parameters and lung injury indicators. Nine core metabolites exhibiting strong correlations (|r| > 0.7, P < 0.01, FDR-corrected) with at least one parameter from either category were selected for comparative visualization. A customized correlation triangle plot was constructed using R software v4.3.0 (R Foundation for Statistical Computing, Austria) with ggplot2 and ggforce packages. Triangle area was scaled proportionally to absolute log_2_ fold change magnitude (range: 0.2–0.7), with upward orientation indicating upregulation (log_2_FC > 0) and downward orientation indicating downregulation (log_2_FC < 0).

### 2.14 Statistical analysis

Data were statistically analyzed and visualized using GraphPad Prism v8.0.2 software (United States). The analysis and visualization of omics data were performed using omicshare tools (https://www.genedenovo.com/). Results are expressed as the mean ± standard deviation (SD). Significant differences were evaluated by one-way analysis of variance (ANOVA) and the Tukey test. A value of P < 0.05 was considered statistically significant.

## 3 Results

### 3.1 Chemical characterization of the ethanol-soluble fraction of *C. pilosula* reveals enrichment in phenolic acids and their derivatives

The ethanol-soluble fraction of *C. pilosula* was analyzed by UPLC-MS in non-targeted full-scan mode, yielding a high-quality total ion chromatogram (Figure 1B) with excellent peak resolution. This enabled the identification of 42 compounds via MS library search (Supplementary Table S3). Complementarily, UPLC-DAD fingerprinting was performed at multiple wavelengths to establish the chemical profile of this fraction (Figure 1C). The characteristic DAD peak clusters at 3–12 min (detected at 320 nm) and 12–18 min (detected at 280 nm) corresponded to the UPLC-MS retention windows of phenolic acids and their derivatives, respectively, confirming them as the dominant chemical categories in the fraction.

Based on combined fingerprint and MS profiling obtained from non-targeted analysis, 12 representative compounds corresponding to the characteristic DAD peaks (Figure 1D) were selected for targeted quantification using external reference standards. Quantitative analysis (Table 1) revealed that phenolic acids constitute the predominant chemical category in the ethanol-soluble fraction of *C. pilosula*, thus, this fraction was designated as *C. pilosula* phenolic preparation (CPPP). Among the 12 quantified compounds, 10 were phenolic acids or their derivatives, collectively accounting for 25.3% (w/w) of the total fraction. Cinnamic acid exhibited the highest content (100,929.17 μg/g), followed by caffeic acid (40,184.91 μg/g), chlorogenic acid (35,877.77 μg/g), neochlorogenic acid (23,033.10 μg/g), and cryptochlorogenic acid (22,883.03 μg/g). Ferulic acid and p-coumaric acid were present at lower concentrations (9,827.08 and 248.10 μg/g, respectively). In addition to these core phenolic acids, CPPP also contained substantial amounts of phenolic derivatives, including the acetylenic glycoside lobetyolin (17,094.00 μg/g), the phenylpropanoid glycoside syringin (336.00 μg/g) and the phenylethanoid glycoside tangshenoside I (1,190.00 μg/g). Furthermore, CPPP contained the terpenoid atractylenolide III (1,162.00 μg/g) and the phenolic acid biosynthetic precursor quinic acid (132.68 μg/g).

**TABLE 1:** Targeted quantification of twelve major compounds in CPPP.

Peak no.	Compound name	Molecular formula	Molecular weight (Da)	Relative content (μg/g)
1	Neochlorogenic acid	C_16_H_18_O_9_	354.31	23033.10
2	Chlorogenic acid	C_16_H_18_O_9_	354.31	35877.77
3	Syringin	C_17_H_24_O_9_	372.37	336.00
4	Cryptochlorogenic acid	C_16_H_18_O_9_	354.31	22883.03
5	Caffeic acid	C_9_H_8_O_4_	180.16	40184.91
6	p-Coumaric acid	C_9_H_8_O_3_	164.16	248.10
7	Cinnamic acid	C_9_H_8_O_2_	148.16	100929.17
8	Ferulic acid	C_10_H_10_O_4_	194.18	9827.08
9	Quinic acid	C_7_H_12_O_6_	192.17	132.68
10	Lobetyolin	C_20_H_28_O_8_	396.43	17094.00
11	Tangshenoside I	C_29_H_42_O_18_	678.64	1190.00
12	Atractylenolide III	C_15_H_20_O_3_	248.32	1162.00

Peaks 1–12 were identified by comparison with external reference standards based on retention time and UV/MS, spectra. Data are expressed as single determination (μg/g dry weight).

### 3.2 CPPP remodels the gut microbiota to enhance barrier-protective and anti-inflammatory metabolites while attenuating harmful microbial metabolites

Based on the hypothesis of this study that CPPP might modulate the gut-lung axis to confer lung protective effects, we investigated whether CPPP-mediated gut microbiota alteration contributed to its protective action against LPS-induced ALI. To this end, the effects of CPPP on gut microbial alterations in mice were investigated by 16S rRNA sequencing. The results (Figure 2A) showed that at the phylum level, the three most dominant core phyla in all four mice groups were Bacillota, Bacteroidota, and Verrucomicrobiota, consistent with the typical phylum-level composition of the gut microbiota in mice as previously reported (Chu and Zhang, 2022). However, their relative abundances showed distinct distribution patterns among the groups (Figure 2A). As an altered Firmicutes/Bacteroidota (F/B) ratio is generally considered as an indicator of gut microbial dysbiosis (Stojanov et al., 2020), we compared the F/B ratio across the four groups. Compared to the normal control (NC) group, pretreatment with CPPP alone (the CPPP group) did not alter the F/B ratio, which remained comparable to that of the NC group (Figure 2B). In contrast, ethanol pretreatment (the EtOH group) significantly (P < 0.001) elevated the F/B ratio compared to the NC group, indicating gut microbial dysbiosis. Pretreatment with both CPPP and ethanol (the EtOH + CPPP group) markedly (P < 0.01) attenuated the ethanol-induced increase in the F/B ratio. These findings suggested that CPPP may protect against ethanol-induced gut microbial dysbiosis by maintaining F/B ratio homeostasis.

**FIGURE 2:**
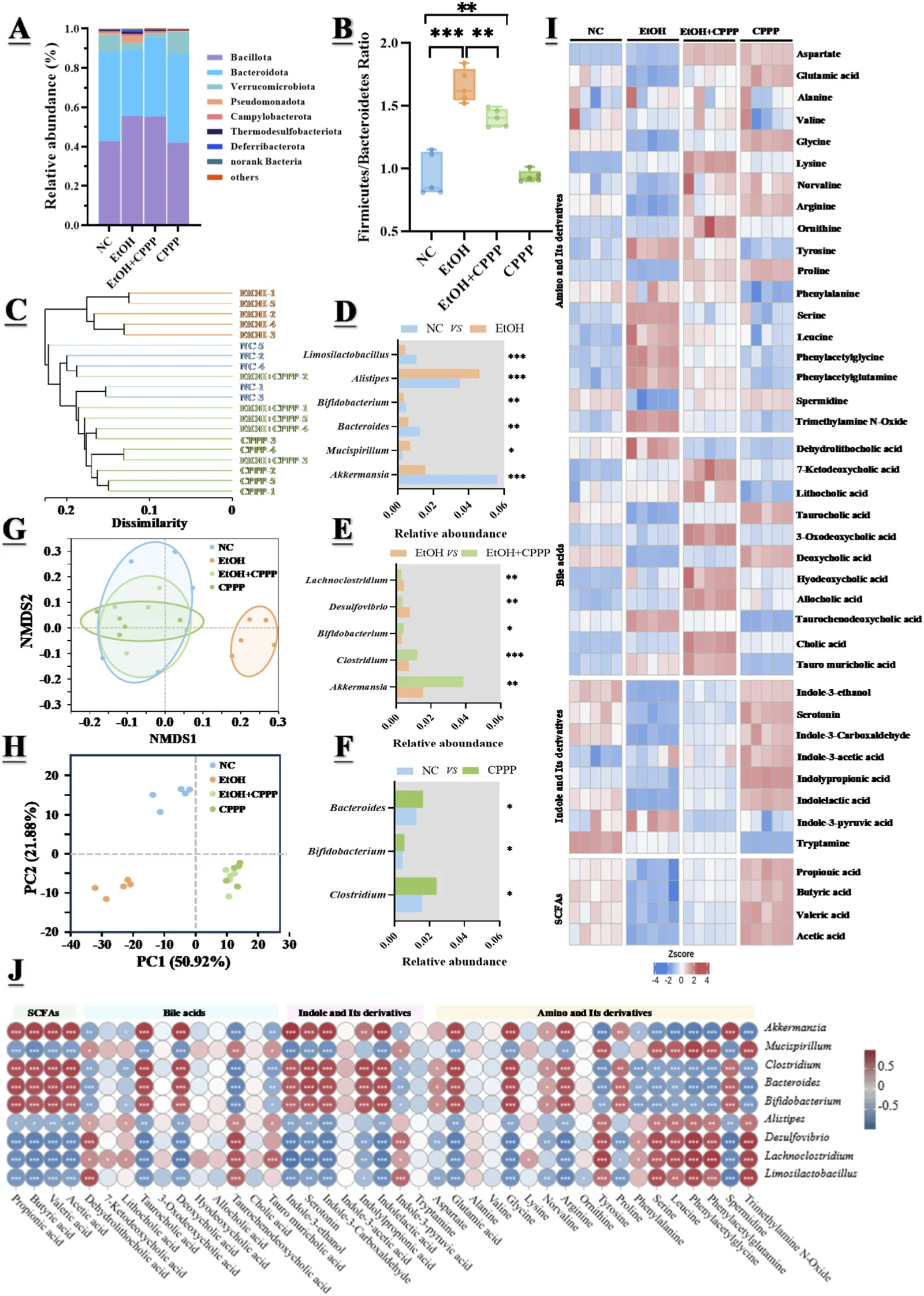
CPPP remodels the gut microbiota to enhance barrier-protective and anti-inflammatory metabolites while attenuating harmful microbial metabolites. **(A)** Changes in the relative abundance of gut microbiota at the phylum level for the NC, EtOH, EtOH + CPPP and CPPP groups (n = 5). **(B)** Firmicutes/Bacteroidetes ratios. **(C)** Hierarchical clustering, **(G)** NMDS and **(H)** PCA analyses of gut microbiota. Differential microbiota between the NC and EtOH groups **(D)**, between the EtOH and EtOH + CPPP. groups **(E)**, and between the NC and CPPP groups **(F)** at genus level. **(I)** Heatmap visualization of fecal metabolite profiles (including amino acids, bile acids, indole and its derivatives, and SCFAs) across NC, EtOH, EtOH + CPPP and CPPP groups, the color gradient (from green to red) represents the Z-score of metabolite abundance: blue indicates relatively low abundance, while red indicates relatively high abundance, reflecting the differential accumulation patterns of metabolites among groups. **(J)** Heatmap visualization of Pearson correlation coefficients between bacterial taxa and fecal metabolite classes (columns: SCFAs, bile acids, indole derivatives, amino acid derivatives). Red circles indicate positive correlations, blue circles indicate negative correlations, with size and intensity reflecting correlation strength. For statistics, * P < 0.05, ** P < 0.01, and *** P < 0.001.

To further analyze the overall differences in gut microbial composition among the groups, alpha and beta diversity analyses were performed at the genus level. Alpha diversity indices Chao1 and Shannon showed no differences among the NC, EtOH + CPPP, and CPPP groups, indicating comparable within-sample microbial richness and evenness across these treatment conditions (Supplementary Figure S1). Thus, this study focused on beta diversity analysis to assess between-group differences in gut microbial community composition. Hierarchical clustering analysis demonstrated that the EtOH group formed a separate clade distinct from the other three groups, which clustered together as a single branch (Figure 2C). This separation was further corroborated by NMDS and PCA ordinations (Figures 2G,H), in which the EtOH group exhibited marked divergence from the other three groups along the primary coordinate axes (NMDS1 and PC1, respectively).

Comparison of key genus-level relative abundances between the EtOH and NC groups (Figure 2D) showed ethanol induced significant gut microbiota dysbiosis, characterized by decrease of beneficial genera (*Limosilactobacillus, Bifidobacterium, Bacteroides* and *Akkermansia*, P < 0.01) and increase of pathogenic genera *Alistipes* (P < 0.001) and *Mucispirillum* (P < 0.05). Comparison between the EtOH and EtOH + CPPP groups (Figure 2E) revealed that CPPP significantly increased the abundance of beneficial genera (*Bifidobacterium, Akkermansia* and *Clostridium*, P < 0.05) and decreased pathogenic genus *Desulfovibrio* (P < 0.01), demonstrating the ability of CPPP to attenuate ethanol-induced gut microbiota dysbiosis. Comparison between the NC and CPPP groups (Figure 2F) revealed that CPPP significantly increased the abundance of beneficial genera (*Clostridium, Bacteroides* and *Bifidobacterium*, P < 0.05). Notably, *Clostridium* and *Bifidobacterium* were consistently enriched by CPPP, whether administered alone or in combination with ethanol. These genera represent key short-chain fatty acids (SCFAs)-producing bacteria that generate butyric acid and other metabolites critical for intestinal barrier maintenance and immune modulation (Fu et al., 2023; O’Callaghan and van Sinderen, 2016). The selective enrichment of these metabolically beneficial genera by CPPP highlighted the unique capacity of CPPP to reshape the gut microbiota toward a metabolically beneficial and barrier-protective state.

Given that gut microbial composition directly shapes the metabolic profile of the host (Fujisaka et al., 2023), we next investigated whether CPPP-mediated microbiota remodeling functionally altered the fecal metabolome. To this end, we quantified the fecal levels of amino acids, indole and its derivatives, bile acids, and SCFAs across all the four mice groups. Integrated metabolomic analysis (Figure 2I) revealed that compared to the NC group, pretreatment with CPPP alone suppressed harmful metabolites (notably trimethylamine N-oxide (TMAO), phenylacetylglycine (PAGly), and phenylacetylglutamine (PAGln)) and bile acids (such as dehydrolithocholic acid and taurochenodeoxycholic acid), while increased beneficial metabolites including spermidine, SCFAs, and tryptophan-derived indoles. Pretreatment with both CPPP and ethanol reversed the ethanol-induced metabolic alterations, specifically TMAO, spermidine, taurochenodeoxycholic acid and butyric acid. These results suggested that CPPP may remodel the metabolome toward a homeostatic profile characterized by enhanced barrier-protective and anti-inflammatory metabolites.

To elucidate the functional basis underlying CPPP-induced gut microbiota remodeling, correlation analysis between gut microbial genera and fecal metabolites were performed (Figure 2J). The CPPP-enriched beneficial genera *Bifidobacterium, Clostridium*, *Bacteroides* and *Akkermansia*, exhibited strong positive correlations with barrier-protective metabolites, including spermidine, SCFAs (butyric acid, propionic acid, acetic acid), and tryptophan-derived indoles (indole-3-ethanol, indolypropionic acid, indole-3-acetic acid, serotonin), while showing negative associations with harmful metabolites (TMAO, PAGly, PAGln, dehydrolithocholic acid, taurochenodeoxycholic acid). Conversely, pathogenic genera *Mucispirillum, Desulfovibrio, Alistipes*, displayed the inverse correlation pattern. These results demonstrated that CPPP may remodel the gut microbiota, thereby promoting the production of barrier-protective and anti-inflammatory metabolites while attenuating harmful microbial metabolites.

### 3.3 CPPP protects intestinal barrier integrity against LPS-induced damage during ALI

The intestinal barrier serves as a critical component of the gut-lung axis, where barrier dysfunction can both amplify local inflammation and propagate systemic inflammatory signals to the lung (Takiishi et al., 2017). To determine whether CPPP-mediated protection against LPS-induced ALI involved preservation of intestinal barrier integrity in mice, H&E staining of jejunal tissue, AB-PAS staining of goblet cells, and immunohistochemical analysis of tight junction proteins were performed. LPS stimulation significantly disrupted intestinal barrier integrity in the NC/LPS + group, as evidenced by reduced villus height (P < 0.001, Figures 3A,B), diminished goblet cell numbers per crypt-villus unit (P < 0.001, Figures 3C,D), and decreased expression of tight junction proteins (ZO-1, Occludin, and Claudin-1) (P < 0.001, Figures 3E–J) compared to the NC/LPS- group. Ethanol pretreatment in the EtOH/LPS + group markedly exacerbated these LPS-induced disruptions compared to the NC/LPS + group (P < 0.001, Figures 3A–J). Conversely, pretreatment with CPPP, whether alone or combined with ethanol, effectively reversed these pathological alterations, restoring villus architecture, goblet cell abundance, and tight junction protein expression (P < 0.001 for EtOH + CPPP/LPS + vs. EtOH/LPS+, P < 0.001 for CPPP/LPS + vs. NC/LPS+, Figures 3A–J). These findings indicated that CPPP may confer intestinal barrier protection against LPS-induced damage during ALI, by preserving villus architecture, maintaining goblet cell-mediated mucus secretion, and restoring tight junction protein expression.

**FIGURE 3:**
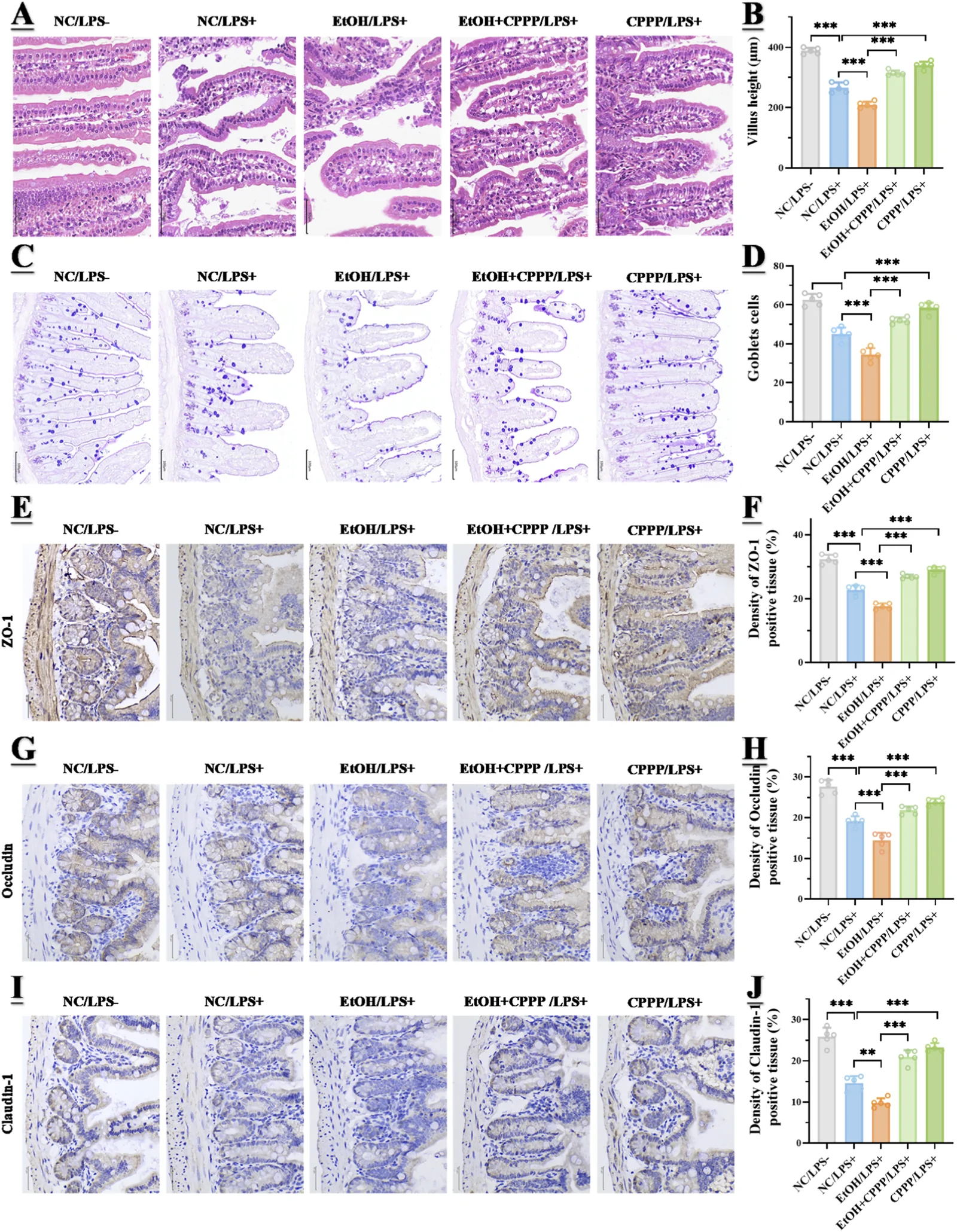
CPPP protects intestinal barrier integrity against LPS-induced damage during ALI. **(A)** Representative H&E-stained sections of jejunal tissue showing histological architecture (original magnification ×400). **(B)** Quantitative analysis of villus length. **(C)** Representative AB-PAS-stained sections illustrating goblet cell distribution (original magnification ×200). **(D)** Quantification of goblet cell number per crypt-villus unit. **(E–J)** Immunohistochemical detection and quantification of tight junction proteins in jejunal tissue: **(E,G,I)** Representative images of the protein expression of ZO-1, Occludin and Claudin-1 (original magnification ×400); **(F,H,J)** Quantitative analysis of the protein expression levels of ZO-1, Occludin and Claudin-1. Data were expressed as mean ± SD (n = 5). For statistics, * P < 0.05, ** P < 0.01, *** P < 0.001, and **** P < 0.0001.

### 3.4 CPPP attenuates LPS-induced ALI by suppressing systemic inflammation

As a critical node in the gut-lung axis, intestinal barrier integrity directly modulates systemic inflammatory status and distal organ susceptibility (Vanuytsel et al., 2021). We therefore investigated whether CPPP-mediated intestinal barrier protection translated into attenuated systemic inflammation and improved pulmonary outcomes in mice with LPS-induced ALI. ELISA analysis (Figures 4A–D) revealed that compared to the NC/LPS + group, pretreatment with CPPP alone markedly attenuated LPS-induced systemic inflammatory response, with decreases in serum levels of pro-inflammatory factors (CRP, TNF-α, and IL-6) and an increase in serum level of IL-10 (P < 0.001). Compared to the EtOH/LPS + group, CPPP pretreatment, whether alone or combined with ethanol, significantly attenuated LPS-induced systemic inflammatory response, decreasing serum levels of CRP, TNF-α, and IL-6 while increasing serum level of IL-10 (P < 0.001).

**FIGURE 4:**
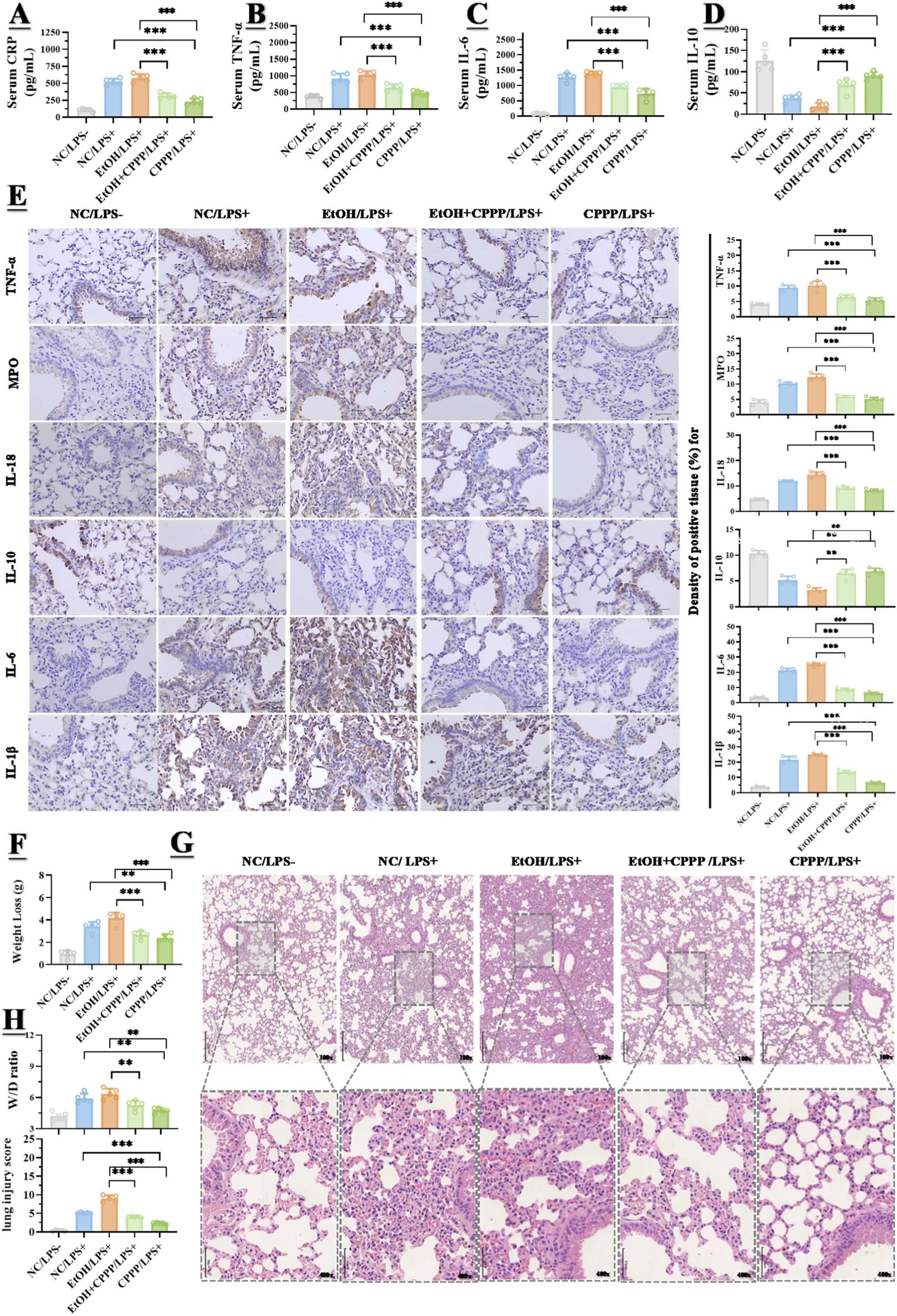
CPPP attenuates LPS-induced ALI by suppressing systemic inflammation. The serum levels of CRP **(A)**, TNF-α **(B)**, IL-6 **(C)**, IL-10 **(D)**. **(E)** Expression of pro-inflammatory cytokines (IL-1β, IL-6, IL-18, TNF-α), neutrophil marker myeloperoxidase (MPO) and anti-inflammatory cytokine IL-10 changes in the lung tissues stained with immunohistochemistry (original magnification ×400). **(F)** Body weight loss. **(G)** Histological changes in the lung tissues stained with H&E (original magnification ×100, ×400). **(H)** Lung W/D ratio and lung injury score. Data were expressed as mean ± SD (n = 5). For statistics, * P < 0.05, ** P < 0.01, *** P < 0.001.

Consistent with the systemic cytokine profile, immunohistochemical analysis of lung tissues (Figure 4E) revealed similar inflammation patterns in the lungs. Compared to the NC/LPS + group, pretreatment with CPPP alone significantly decreased levels of pro-inflammatory cytokines (IL-1β, IL-6, IL-18, TNF-α) and the neutrophil infiltration marker MPO (P < 0.001) in lung tissues, while increased the IL-10 level (P < 0.01). Compared to the EtOH/LPS + group, CPPP pretreatment, whether alone or combined with ethanol, significantly suppressed pro-inflammatory cytokines and MPO in lung tissues while increasing the IL-10 level (P < 0.001 for pro-inflammatory cytokines; P < 0.01 for IL-10).

These protective effects were further corroborated by physiological outcomes of the lungs. Compared to the NC/LPS + group, pretreatment with CPPP alone significantly attenuated LPS-induced weight loss (P < 0.01, Figure 4F), reduced pulmonary edema as assessed by lung wet/dry weight ratio (P < 0.01, Figure 4H), and ameliorated histopathological damage as quantified by lung injury scores (P < 0.001, Figure 4H). Compared to the EtOH/LPS + group, CPPP pretreatment, whether alone or combined with ethanol, significantly attenuated LPS-induced weight loss (P < 0.001, Figure 4F), reduced pulmonary edema (P < 0.01, Figure 4H), and ameliorated histopathological damage (P < 0.001, Figure 4H). Histopathological examination (Figure 4G) confirmed these quantitative findings that the NC/LPS+ and EtOH/LPS + groups displayed characteristic features of ALI, including alveolar collapse, vascular congestion, and neutrophil cell infiltration, whereas CPPP pretreatment preserved alveolar-capillary barrier integrity and attenuated neutrophil cell infiltration in the EtOH + CPPP/LPS+ and CPPP/LPS + groups. Together, these results suggested that CPPP may attenuate LPS-induced ALI by suppressing systemic inflammation, consistent with its protective effects on intestinal barrier integrity within the gut-lung axis.

### 3.5 CPPP strengthens the gut-lung axis by attenuating endotoxin translocation and preserving systemic metabolic homeostasis

The interaction between gut microbiota and their metabolites extends to the systemic circulation through the gut-lung axis, with serum metabolites serving as direct indicators of host systemic metabolic status (Fujisaka et al., 2023). As CPPP-mediated protection of intestinal barrier integrity and suppression of systemic inflammation within the gut-lung axis would be expected to alter systemic metabolic profiles and reduce endotoxin translocation, we quantified serum levels of metabolites and LPS to validate these mechanistic consequences. Principal component analysis (PCA) of serum metabolites (Figure 5A) revealed distinct clustering of the NC/LPS–group apart from the LPS-stimulated groups. Notably, the EtOH + CPPP/LPS+ and CPPP/LPS + groups formed a tight cluster that was clearly separated from the EtOH/LPS + group.

**FIGURE 5:**
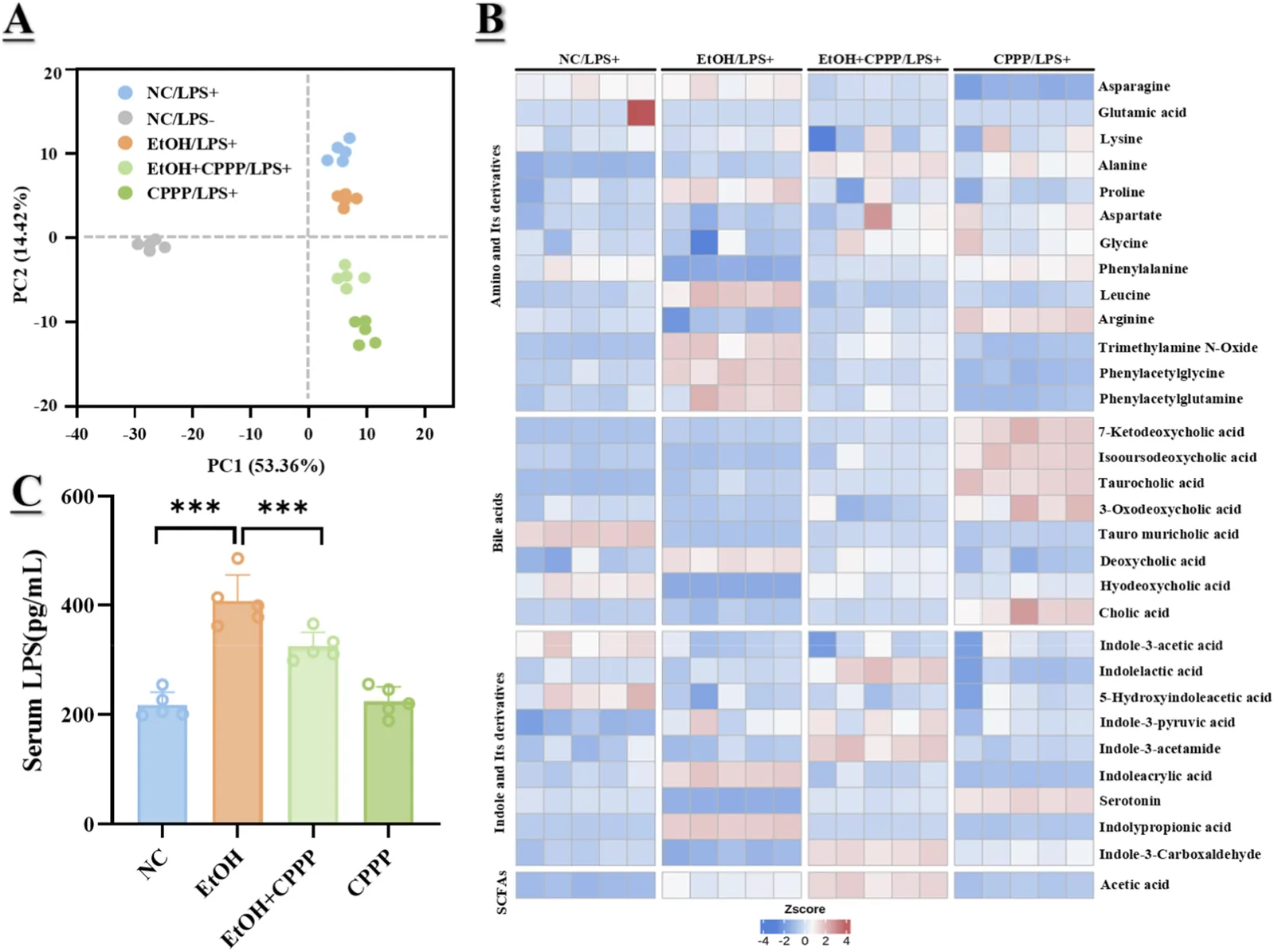
CPPP strengthens the gut-lung axis by attenuating endotoxin translocation and preserving systemic metabolic homeostasis. **(A)** PCA analysis of serum metabolites among all five groups. **(B)** Heatmap visualization of serum metabolite profiles (including amino acids, bile acids, indole and its derivatives, and SCFAs) across the four LPS-stimulated treatment groups, the color gradient from blue to red represents the Z-score of metabolite abundance from low to high. **(C)** Quantification of the serum LPS levels across the four LPS-stimulated treatment groups. Data were expressed as mean ± SD (n = 5). For statistics, * P < 0.05, ** P < 0.01, *** P < 0.001.

To further characterize metabolite alterations under LPS-stimulated conditions, a heatmap of differentially abundant metabolites among the four LPS-stimulated groups (Figure 5B) showed that pretreatment with both CPPP and ethanol elicited pronounced metabolic reprogramming compared to the EtOH/LPS + group. Specifically, CPPP restored ethanol-induced gut microbiota-derived amino acid metabolites (e.g., TMAO, PAGly, and PAGln), bile acids (e.g., deoxycholic acid and hyodeoxycholic acid), and indole derivatives (e.g., indoleacrylic acid, indolepropionic acid, and serotonin) toward the levels of the NC/LPS + group, while enhancing acetic acid abundance. The CPPP/LPS + group exhibited a metabolic profile comparable to that of the NC/LPS + group, supporting the ability of CPPP to maintain metabolic homeostasis under systemic inflammatory conditions.

Compared to the EtOH/LPS + group, pretreatment with both CPPP and ethanol reversed the ethanol-induced elevation in serum LPS levels (P < 0.001; Figure 5C). The CPPP/LPS + group showed serum LPS levels comparable to that of the NC/LPS + group. These findings indicated that CPPP may strengthen the gut-lung axis by preserving intestinal barrier integrity, reducing endotoxin translocation, and thereby maintaining systemic metabolic homeostasis under inflammatory challenge.

### 3.6 CPPP attenuates LPS-induced ALI through a metabolite-mediated gut-lung axis

To further validate the functional role of metabolites as mediators within the gut-lung axis, correlations were analyzed between fecal metabolites and intestinal barrier markers, and between serum metabolites and lung injury indicators. As shown in Figure 6A, fecal metabolites including TMAO (P < 0.001), PAGly (P < 0.01) and PAGln (P < 0.05), as well as bile acids including dehydrolithocholic acid (P < 0.001) and taurochenodeoxycholic acid (P < 0.001), demonstrated significant negative correlations with intestinal barrier markers villus height, goblet cell numbers, and expression of tight junction proteins (ZO-1, Occludin, Claudin-1). Conversely, the amino acid derivatives spermidine (P < 0.001), bile acids including deoxycholic acid (P < 0.01) and taurocholic acid (P < 0.001), indole derivatives such as serotonin (P < 0.01), indole-3-ethanol (P < 0.01), indolypropionic acid (P < 0.001) and indolelactic acid (P < 0.001), SCFAs such as butyric acid (P < 0.001), propionic acid (P < 0.001) and acetic acid (P < 0.001) demonstrated significant positive correlations with intestinal barrier markers villus height, goblet cell numbers, and expression of tight junction proteins (ZO-1, Occludin, Claudin-1). The seven fecal metabolites TMAO, PAGly, PAGln, spermidine, serotonin, indole-3-ethanol and butyric acid were precisely those modulated by CPPP, suggesting that these gut-derived metabolites serve as functional mediators linking CPPP-induced microbial community changes to preserved intestinal barrier architecture and function.

**FIGURE 6:**
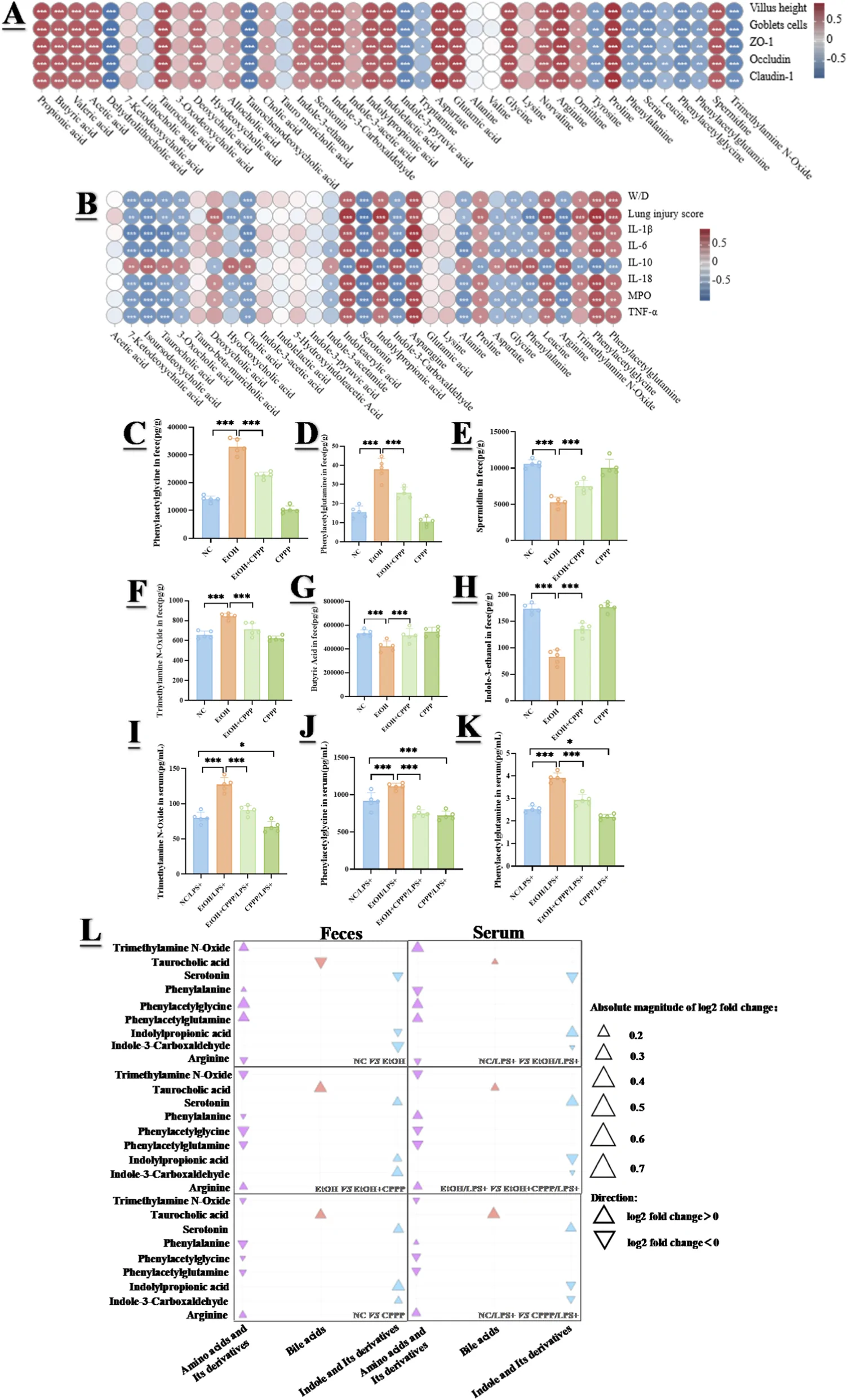
CPPP attenuates LPS-induced ALI through a metabolite-mediated gut-lung axis. **(A)** Correlation analysis between fecal differential metabolites and intestinal barrier integrity. **(B)** Correlation between serum differential metabolites and lung injury indicators. **(C–K)** Statistical quantification of key differential metabolites in feces and serum across the four LPS-stimulated treatment groups. **(L)** Comparative analysis of nine core metabolites shared between feces and serum, displaying strong correlations (|r|>0.7, P < 0.01) with intestinal barrier integrity parameters or lung injury indicators. Triangle size represents absolute log_2_ fold change magnitude (0.2–0.7), orientation indicates directionality (upward: log_2_FC > 0; downward: log_2_FC < 0), and colors denote metabolic pathway classification. Data were expressed as mean ± SD (n = 5). For statistics, * P < 0.05, ** P < 0.01, *** P < 0.001.

As shown in Figure 6B, TMAO (P < 0.05), serum metabolites PAGly (P < 0.001), PAGln (P < 0.01) and indolypropionic acid (P < 0.01), exhibited strong positive correlations with lung injury indicators wet/dry weight ratio, lung injury score, MPO activity, and pro-inflammatory cytokines (IL-1β, IL-6, TNF-α), with the exception that TMAO showed no significant correlation with TNF-α. All four serum metabolites were negatively associated with anti-inflammatory IL-10. Conversely, serum metabolites cholic acid (P < 0.01), taurocholic acid (P < 0.01) and serotonin (P < 0.001), demonstrated significant negative correlations with lung injury indicators wet/dry weight ratio, lung injury score, MPO activity, and pro-inflammatory cytokines (IL-1β, IL-6, TNF-α), alongside positive associations with IL-10. The four serum metabolites TMAO, PAGly, PAGln, and serotonin, were precisely those modulated by CPPP, suggesting that these circulating metabolites serve as functional mediators of lung protective effects of CPPP within the gut-lung axis.

To validate the differential metabolites identified by untargeted metabolomic analysis and functionally implicated by correlation analyses, targeted quantification of key metabolites in feces and serum was performed across the four LPS-stimulated groups (Figures 6C–K). Pretreatment with both CPPP and ethanol significantly (P < 0.001) reversed the ethanol-induced increases in the fecal levels of PAGly, PAGln and TMAO and decreases in the fecal levels of spermidine, butyric acid and indole-3-ethanol, restoring these metabolites levels toward that of the NC group (Figures 6C–H). The CPPP/LPS + group exhibited the fecal levels of these metabolites comparable to that of the NC/LPS + group. These findings quantitatively confirmed the correlations between the fecal metabolites and intestinal barrier markers shown in Figure 6A. Pretreatment with both CPPP and ethanol significantly (P < 0.001) reversed the ethanol-induced increases in the serum levels of PAGly, PAGln and TMAO, restoring these metabolites levels toward that of the NC group (Figures 6I–K). The CPPP/LPS + group exhibited the serum levels of these metabolites comparable to that of the NC/LPS + group. These findings quantitatively confirmed the correlations between the serum metabolites and lung injury indicators shown in Figure 6B.

To identify metabolites that functionally bridge the gut-lung axis across compartments, we performed a comparative analysis of nine metabolites detected in both feces and serum that displayed strong correlations (|r|>0.7, P < 0.01) with intestinal barrier markers (fecal) or lung injury indicators (serum) across all groups (Figure 6L). Comparison between the NC/LPS+ and CPPP/LPS + groups showed that pretreatment with CPPP alone reduced fecal levels of TMAO, PAGly, and PAGln, metabolites strongly correlated with compromised barrier integrity, and reduced their systemic levels in serum, where they correlated with lung injury severity. In addition, pretreatment with CPPP alone increased fecal and serum levels of serotonin, a metabolite positively associated with barrier markers (fecal) and negatively associated with lung injury (serum). These results demonstrated that CPPP may attenuate LPS-induced ALI through a metabolite-mediated gut-lung axis. By reducing intestinal generation of harmful metabolites (TMAO, PAGly, PAGln), CPPP limited their systemic accumulation in serum, thereby attenuating lung injury. Concomitantly, CPPP increased intestinal production of serotonin, enhancing circulating levels of this protective metabolite to support lung homeostasis.

## 4 Discussion

Polysaccharides isolated from aqueous extracts of *C. pilosula* have been reported to attenuate LPS-induced ALI in mice by suppressing pro-inflammatory cytokines and chemokines (Gong et al., 2022), an effect mediated at least in part through the enhancement of intestinal barrier integrity (Fu et al., 2018; Zhou et al., 2025). However, these polysaccharides precipitate during traditional alcoholic preparation of *C. pilosula* and are therefore largely absent from the consumed extract. This study showed that the ethanol-soluble fraction possessed a phytochemical profile distinct from that of aqueous preparations, with phenolic acids and their derivatives accounting for 25.3% of this fraction. The novelty of this study lied in demonstrating that this phenolic-enriched preparation (designated as CPPP) attenuated LPS-induced ALI in mice through the gut-lung axis. Indeed, several of its major constituents are known to support this mechanism. Cinnamic acid and its hydroxylated derivatives (caffeic acid, ferulic acid and p-coumaric acid) have been demonstrated to enhance intestinal barrier function by upregulating expression of tight junction proteins (ZO-1, Occludin, Claudin-1), regulating gut microbiota and reducing pro-inflammatory cytokines (Wang Z. Y. et al., 2023). Chlorogenic acid and its isomers (neochlorogenic acid and cryptochlorogenic acid) preserve intestinal barrier integrity through similar mechanisms, including suppression of pro-inflammatory cytokines, modulation of gut microbial composition and metabolism, and promotion of mucosal repair (Yuan et al., 2025). Lobetyolin, syringin, and tangshenoside I serve as characteristic bioactive markers of *C. pilosula*, lobetyolin being the primary pharmacopoeial marker, and the latter two essential for quality control (Dong et al., 2023; Li et al., 2025). These three constituents contribute to inhibition of pulmonary inflammatory responses through immune regulation (Dong et al., 2023; Li et al., 2025).

The gut microbiota serves as a primary mediator of the gut-lung axis, through which dietary constituents modulate host physiology (Vanuytsel et al., 2021). This study showed that compared to the normal control, CPPP enriched SCFAs-producing bacteria *Bifidobacterium, Akkermansia* and *Clostridium*. *Bifidobacterium* produces acetic acid and lactic acid, maintaining an acidic luminal environment that inhibits pathogen colonization while directly stimulating expression of tight junction proteins in intestinal epithelial cells (O’Callaghan and van Sinderen, 2016). *Akkermansia*, a prominent mucin-degrading bacterium, generates propionic acid and reinforces the mucus layer thickness, thereby enhancing the physical intestinal barrier against luminal antigens (Mo et al., 2024). *Clostridium* is a major producer of butyric acid, which serves as the primary energy source for colonocytes and induces expression of tight junction proteins (Shin et al., 2024; Singh et al., 2022). These findings suggested that CPPP remodeled the gut microbiota toward a barrier-protective profile, thereby engaging the gut-lung axis to modulate host physiology and extend local intestinal protection to systemic anti-inflammatory effects in the lung.

Furthermore, this study showed that CPPP increased the production of beneficial metabolites, including SCFAs (e.g., butyric acid, propionic acid, acetic acid) and tryptophan-derived indoles (e.g., indole-3-propionic acid, serotonin), while reducing potentially deleterious microbiota-derived amino acid metabolites (e.g., TMAO, PAGly, PAGln). The elevated tryptophan-derived indole derivatives suggested CPPP enhanced microbial tryptophan metabolism, which activates the aryl hydrocarbon receptor (AhR) signaling pathway critical for intestinal immune homeostasis (Hu et al., 2025; Scott et al., 2020). The reduced levels of TMAO, PAGly, and PAGln, metabolites established as causal contributors to inflammatory diseases (Caradonna et al., 2025), suggested CPPP suppressed pro-inflammatory metabolic pathways. Analysis of correlation between fecal metabolites and intestinal barrier markers revealed that barrier-preserving metabolites, particularly SCFAs (butyric acid, propionic acid, acetic acid) and tryptophan-derived indoles (e.g., indole-3-propionic acid, serotonin), exhibited strong positive correlations with intestinal barrier markers, whereas TMAO, PAGly, and PAGln showed negative correlations. The CPPP-mediated enhancement of barrier-preserving metabolites and suppression of barrier-disruptive ones, were consistent with the CPPP-mediated preservation of intestinal barrier integrity, including the maintenance of villus architecture, goblet cell populations, and expression of ZO-1, Occludin, and Claudin-1. The preservation of goblet cells is particularly important, as these cells secrete mucins that constitute the physical mucus barrier and produce regulatory factors that modulate immune responses (Gustafsson and Johansson, 2022).

The gut-lung axis, through which intestinal homeostasis critically shapes pulmonary immunity, has emerged as a key pathway in respiratory disease pathogenesis (Enaud et al., 2020; Liu et al., 2026). The intestinal barrier constitutes the primary anatomical and functional substrate of this axis, serving as the gateway through which gut-derived signals influence systemic and pulmonary health (Sun et al., 2026). In this study, CPPP remodeled the gut microbiota to promote anti-inflammatory metabolites (e.g., butyric acid) and suppress pro-inflammatory metabolites (e.g., TMAO), thereby enhancing intestinal barrier integrity. This structural enhancement translated into a balanced systemic immune profile, as evidenced by CPPP-mediated decrease of serum levels of LPS, pro-inflammatory factors (CRP, TNF-α, IL-6), and increase of serum level of the anti-inflammatory cytokine IL-10. CPPP also exerted distal immunomodulatory effects on lung tissue, reducing local levels of the neutrophil infiltration marker MPO, pro-inflammatory cytokines (IL-1β, IL-6, IL-18) and increasing local level of IL-10. Connecting these serum metabolite-driven events to pulmonary outcomes, correlation analysis revealed that CPPP-modulated serum metabolites, particularly butyric acid and serotonin, exhibited significant negative correlations with lung injury indicators, whereas TMAO, PAGly, and PAGln showed positive correlations. These coordinated microbiota-driven metabolite shifts established a mechanistic link along the gut-lung axis, connecting preserved intestinal barrier function to attenuated lung injury and positioning CPPP-mediated barrier enhancement as the initiating event that propagates protective signals from the gut to the lung.

From a translational perspective, these findings address a critical knowledge gap concerning the health implications of traditional alcohol-steeped *C. pilosula* preparations. This study demonstrated that pretreatment with *C. pilosula* Liquor (i.e., CPPP + ethanol) attenuated LPS-induced ALI by counteracting ethanol-induced intestinal barrier disruption and systemic inflammatory signaling. Similarly, pretreatment with Zhuyeqing Liquor, an alcohol-steeped traditional Chinese medicine preparation rich in flavonoids, iridoids, terpenoids, and phenolic acids, has been shown to reverse ethanol-induced biochemical abnormalities, histopathological damage, and hepatic TNF-α expression in a mouse model of acute liver injury (Gao et al., 2013). This alignment across distinct alcohol-based traditional Chinese medicine formulations underscores a broader pharmacological principle with significant public health relevance, given the widespread consumption of medicinal liquors in East Asian populations. Crucially, these benefits are observed exclusively within the context of moderate controlled alcohol exposure and should not be interpreted as an endorsement of alcohol consumption for health promotion.

This study has several limitations. First, the chemical complexity of CPPP precluded definitive attribution of the observed effects to individual compounds. However, synergistic interactions among phenolic acids and their derivatives might contribute to the overall protective effects of CPPP against LPS-induced ALI. Future studies should employ bioactivity-guided fractionation and *in vivo* validation of purified individual compounds to pinpoint the key active constituents. Second, while the 30-day pretreatment protocol was designed to mirror traditional consumption practices, it precludes determination of the minimal effective exposure duration and precludes characterization of dose-response dynamics for CPPP-mediated protective effects. Future studies should systematically evaluate shorter pretreatment intervals and multiple dose schedules to establish the optimal dosing strategy and the onset of protective effects. Third, while correlation analyses in this study suggested causal relationships between specific bacterial genera, metabolites, and physiological outcomes, definitive causal inference required interventional studies employing microbiota depletion or fecal microbiota transplantation. Future studies should perform fecal microbiota transplantation from CPPP-treated donors into germ-free or antibiotic-treated mice, alongside targeted metabolite supplementation, to establish causality along the gut-lung axis.

## 5 Conclusion

This study provided the first experimental evidence for validating the traditional alcohol-steeping preparation of C. *pilosula* (i.e., *C. pilosula* Liquor). It demonstrated that its phenolic-enriched ethanol-soluble fraction (termed as CPPP) significantly attenuated LPS-induced ALI in mice by remodeling gut microbiota, reprogramming key metabolites and suppressing pro-inflammatory factors along the gut-lung axis. Specifically, CPPP maintained gut microbiota homeostasis, preserved intestinal barrier integrity, and subsequently attenuated both pulmonary and systemic inflammation. This study provides scientific rationale for the use of alcohol-steeping as a bioactive extraction methodology. The identification of the phenolic acid-microbiota-metabolite regulatory cascade as critical mediators of pulmonary protection offers novel targets for the development of microbiome-targeted interventions in respiratory diseases.

## Data Availability

The original contributions presented in the study are publicly available. This data can be found in the NCBI repository, accession number PRJNA1538002; available at: https://www.ncbi.nlm.nih.gov/sra/?term=PRJNA1538002.

## References

[B1] Ashique S. De Rubis G. Sirohi E. Mishra N. Rihan M. Garg A. (2022). Short chain fatty acids: fundamental mediators of the gut-lung axis and their involvement in pulmonary diseases. Chem. Biol. Interact. 368, 110231. 10.1016/j.cbi.2022.110231 36288778

[B2] Bao X. Sun Y. Xu H. Han R. Wan M. Wang Y. (2026). The mechanism of *Codonopsis pilosula* in treating chronic obstructive pulmonary disease via exploring network pharmacology and multi-omics. J. Ethnopharmacol. 359, 121082. 10.1016/j.jep.2025.121082 41448367

[B3] Bolyen E. Rideout J. R. Dillon M. R. Bokulich N. A. Abnet C. C. Al-Ghalith G. A. (2019). Reproducible, interactive, scalable and extensible microbiome data science using QIIME 2. Nat. Biotechnol. 37, 852–857. 10.1038/s41587-019-0209-9 31341288 PMC7015180

[B4] Caradonna E. Abate F. Schiano E. Paparella F. Ferrara F. Vanoli E. (2025). Trimethylamine-N-Oxide (TMAO) as a rising-star metabolite: implications for human health. Metabolites 15, 220. 10.3390/metabo15040220 40278349 PMC12029716

[B5] China N.P.C.o.t.P.s.R.o (2020). Pharmacopoeia of the People's Republic of China. 2020 Edition. Beijing, China: China Medical Science Press.

[B6] Chu M. Zhang X. (2022). Bacterial atlas of mouse gut microbiota. Cell Microbiol. 2022, 5968814–5968821. 10.1155/2022/5968814

[B7] Dong J. Na Y. Hou A. Zhang S. Yu H. Zheng S. (2023). A review of the botany, ethnopharmacology, phytochemistry, analysis method and quality control, processing methods, pharmacological effects, pharmacokinetics and toxicity of codonopsis radix. Front. Pharmacol. 14, 1162036. 10.3389/fphar.2023.1162036 37089919 PMC10117688

[B8] Enaud R. Prevel R. Ciarlo E. Beaufils F. Wieërs G. Guery B. (2020). The gut-lung axis in health and respiratory diseases: a place for inter-organ and inter-kingdom crosstalks. Front. Cell Infect. Microbiol. 10, 9. 10.3389/fcimb.2020.00009 32140452 PMC7042389

[B9] Fu Y. P. Feng B. Zhu Z. K. Feng X. Chen S. F. Li L. X. (2018). The polysaccharides from *Codonopsis pilosula* modulates the immunity and intestinal microbiota of cyclophosphamide-treated immunosuppressed mice. Molecules 23, 1801. 10.3390/molecules23071801 30037030 PMC6100181

[B10] Fu Y. Lyu J. Wang S. (2023). The role of intestinal microbes on intestinal barrier function and host immunity from a metabolite perspective. Front. Immunol. 14, 1277102. 10.3389/fimmu.2023.1277102 37876938 PMC10591221

[B11] Fujisaka S. Watanabe Y. Tobe K. (2023). The gut microbiome: a core regulator of metabolism. J. Endocrinol. 256, e220111. 10.1530/JOE-22-0111 36458804 PMC9874984

[B12] Gao H. Y. Huang J. Wang H. Y. Du X. W. Cheng S. M. Han Y. (2013). Protective effect of zhuyeqing liquor, a Chinese traditional health liquor, on acute alcohol-induced liver injury in mice. J. Inflamm. (Lond) 10, 30. 10.1186/1476-9255-10-30 24090365 PMC3851207

[B13] Gong Z. Zhang S. Gu B. Cao J. Mao W. Yao Y. (2022). Codonopsis pilosula polysaccharides attenuate escherichia coli-induced acute lung injury in mice. Food Funct. 13, 7999–8011. 10.1039/d2fo01221a 35818994

[B14] Gustafsson J. K. Johansson M. E. V. (2022). The role of goblet cells and mucus in intestinal homeostasis. Nat. Rev. Gastroenterol. Hepatol. 19, 785–803. 10.1038/s41575-022-00675-x 36097076

[B15] Hu W. Garrison C. Prasad R. Boulton M. E. Grant M. B. (2025). Indole metabolism and its role in diabetic macrovascular and microvascular complications. Am. Heart J. Plus 53, 100532. 10.1016/j.ahjo.2025.100532 40230659 PMC11995707

[B16] Li M. H. He Y. H. Chen C. B. Liu L. Xu J. Cao J. H. (2025). Ecological factors drive the accumulation of active components in *Codonopsis pilosula* . Phyt. J. Exp. Bot. 94, 2575–2591. 10.32604/phyton.2025.064518

[B17] Liu J. Hong W. Sun Z. Zhang S. Xue C. Dong N. (2026). The gut-lung axis: effects and mechanisms of gut microbiota on pulmonary diseases. Front. Immunol. 16, 16–2025. 10.3389/fimmu.2025.1693964 41562083 PMC12812986

[B18] Lu S. Gu W. Ma Q. Tian R. Qiu R. Ma L. (2023). Extraction, structural characterization, and biological activities of a new glucan from Codonopsis pilosula. Sci. Rep. 13, 4504. 10.1038/s41598-023-31660-2 36934161 PMC10024767

[B19] Mikawa K. Nishina K. Takao Y. Obara H. (2003). ONO-1714, a nitric oxide synthase inhibitor, attenuates endotoxin-induced acute lung injury in rabbits. Anesth. Analg. 97, 1751–1755. 10.1213/01.Ane.0000086896.90343.13 14633554

[B20] Mo C. Lou X. Xue J. Shi Z. Zhao Y. Wang F. (2024). The influence of *Akkermansia muciniphila* on intestinal barrier function. Gut Pathog. 16, 41. 10.1186/s13099-024-00635-7 39097746 PMC11297771

[B21] Mowery N. T. Terzian W. T. H. Nelson A. C. (2020). Acute lung injury. Curr. Probl. Surg. 57, 100777. 10.1016/j.cpsurg.2020.100777 32505224

[B22] O’Callaghan A. A. van Sinderen D. (2016). Bifidobacteria and their role as members of the human gut microbiota. Front. Microbiol. 7, 925. 10.3389/fmicb.2016.00925 27379055 PMC4908950

[B23] Scott S. A. Fu J. Chang P. V. (2020). Microbial tryptophan metabolites regulate gut barrier function via the aryl hydrocarbon receptor. PNAS 117, 19376–19387. 10.1073/pnas.2000047117 32719140 PMC7431026

[B24] Shin J. H. Tillotson G. MacKenzie T. N. Warren C. A. Wexler H. M. Goldstein E. J. C. (2024). Bacteroides and related species: the keystone taxa of the human gut microbiota. Anaerobe 85, 102819. 10.1016/j.anaerobe.2024.102819 38215933

[B25] Singh V. Lee G. Son H. Koh H. Kim E. S. Unno T. (2022). Butyrate producers, “the sentinel of gut”: their intestinal significance with and beyond butyrate, and prospective use as microbial therapeutics. Front. Microbiol. 13, 1103836. 10.3389/fmicb.2022.1103836 36713166 PMC9877435

[B26] Stojanov S. Berlec A. Strukelj B. (2020). The influence of probiotics on the firmicutes/bacteroidetes ratio in the treatment of obesity and inflammatory bowel disease. Microorganisms 8, 1715. 10.3390/microorganisms8111715 33139627 PMC7692443

[B27] Sun N. Liu W. Li J. Zhang D. Liu X. Liu J. (2026). Composition, function, and detection methods of the intestinal barrier. J. Nutr. Biochem. 149, 110179. 10.1016/j.jnutbio.2025.110179 41232652

[B28] Takiishi T. Fenero C. I. M. Camara N. O. S. (2017). Intestinal barrier and gut microbiota: shaping our immune responses throughout life. Tissue Barriers 5, e1373208. 10.1080/21688370.2017.1373208 28956703 PMC5788425

[B29] Tang J. Xu L. Zeng Y. Gong F. (2021). Effect of gut microbiota on LPS-induced acute lung injury by regulating the TLR4/NF-kB signaling pathway. Int. Immunopharmacol. 91, 107272. 10.1016/j.intimp.2020.107272 33360370

[B30] Vanuytsel T. Tack J. Farre R. (2021). The role of intestinal permeability in gastrointestinal disorders and current methods of evaluation. Front. Nutr. 8, 717925. 10.3389/fnut.2021.717925 34513903 PMC8427160

[B31] Verma A. Bhagchandani T. Rai A. Nikita S. U. K. Bhavesh N. S. (2024). Short-chain fatty acid (SCFA) as a connecting link between microbiota and gut-lung axis-a potential therapeutic intervention to improve lung health. ACS Omega 9, 14648–14671. 10.1021/acsomega.3c05846 38585101 PMC10993281

[B32] Wang L. Cai Y. Garssen J. Henricks P. A. J. Folkerts G. Braber S. (2023a). The bidirectional gut-lung axis in chronic obstructive pulmonary disease. Am. J. Respir. Crit. Care Med. 207, 1145–1160. 10.1164/rccm.202206-1066TR 36883945 PMC10161745

[B33] Wang Z. Y. Yin Y. Li D. N. Zhao D. Y. Huang J. Q. (2023b). Biological activities of p-hydroxycinnamic acids in maintaining gut barrier integrity and function. Foods 12, 2636. 10.3390/foods12132636 37444374 PMC10340781

[B34] Yi S. Zhuang X. Luo L. Fu L. Dong Z. Wang K. (2026). Gut microbiota and metabolites in acute lung injury: mechanisms and therapeutic perspectives. Resp. Res. 27, 82. 10.1186/s12931-026-03525-5 41580828 PMC12914935

[B35] Yuan Q. Liu C. Zhang Z. Chen F. Xiao Q. Zhang L. (2025). Pharmacological advances of the chlorogenic acids family: current insights and future research directions. Front. Pharmacol. 16, 1613048. 10.3389/fphar.2025.1613048 41142236 PMC12549615

[B36] Zhou J. Yang Q. Wei W. Huo J. Wang W. (2025). *Codonopsis pilosula* polysaccharide alleviates ulcerative colitis by modulating gut microbiota and SCFA/GPR/NLRP3 pathway. J. Ethnopharmacol. 337, 118928. 10.1016/j.jep.2024.118928 39393557

[B37] Zou Y. Yan H. Li C. Wen F. Jize X. Zhang C. (2023). A pectic polysaccharide from codonopsis pilosula alleviates inflammatory response and oxidative stress of aging mice via modulating intestinal microbiota-related gut-liver axis. Antioxidants (Basel) 12, 1781. 10.3390/antiox12091781 37760084 PMC10525188

