# Supplementary material for: Phenolic-enriched ethanol-soluble fraction of Codonopsis pilosula attenuates LPS-induced acute lung injury in mice via the gut-lung axis
Source: Front Pharmacol. 2026 Oct 7;17:1863500. doi: 10.3389/fphar.2026.1863500 (PMC13683668; doi:10.3389/fphar.2026.1863500)
Supplement: Supplementary file 1 [file Supplementaryfile1.docx]

**Supplementary Materials**

**for**

**Phenolic-enriched ethanol-soluble fraction of *Codonopsis pilosula* attenuates LPS-induced acute lung injury in mice via the gut-lung axis**

**Jiao Li^a^****^1^, Qian Xiao^a1^,** **Yi Ran Sun^b^, Si Yu Zhang^a^, Xun Gou^a^, Jie Xie^a^, Ying Li^c,d^, Jia Hui Yuan^a^, Ling Ling Zeng^a^, Guang Bin Meng^a^, Jian Xin Chai^c,d*^, Xiao Yu Zhang^a^****^*^**

a, College of Life Sciences, Sichuan Normal University, Chengdu, 610101, P. R. China

b, School of Pharmacy, Chengdu Medical College, Chengdu, 610500, P. R. China

c, Shanxi Zhendong Wuhe Yi Yang Tang Co., Ltd., Changzhi, 047100, P. R. China

d, Shanxi Key Laboratory of Medicinal and Edible Homology Functional Food, Chang zhi, 047100, P. R. China

**legends:**

**1) Table S1 Composition of experiment diet**

**2) Table S2 Energy ratio of experiment diet**

**3) Table S3 Identification and quantification of major compounds in CPPP by** **non-targeted metabolomic analysis**

**4) Figure S1 Alpha diversity of gut microbiota across all groups. (A) Chao1 index. (B) Shannon index.**

| **Table S1 Composition of experiment diet** | | |
| --- | --- | --- |
| **Compounds** | **gm** | **Kcal** |
| Casein, 80 mesh | 200 | 800 |
| L-cystine | 3 | 12 |
| corn starch | 315 | 1260 |
| maltodextrin 10 | 35 | 140 |
| sucrose | 350 | 1400 |
| Cellulose BW200 | 50 | 0 |
| soybean oil | 25 | 225 |
| lard | 20 | 180 |
| Mixed Minerals S10026 | 10 | 0 |
| CaHPO₄ | 13 | 0 |
| CaCO₃ | 5.5 | 0 |
| Potassium citrate, 1 H₂O | 16.5 | 0 |
| Multivitamin V10001 | 10 | 40 |
| choline bitartrate | 2 | 0 |
| FD&C Yellow Dye #5 | 0.05 | 0 |
| total | 1055.05 | 4057 |

| **Table S2 Energy ratio of experiment diet** | | |
| --- | --- | --- |
| **Compounds** | **gm (%)** | **Kcal (%)** |
| protein | 19.2 | 20 |
| carbohydrate | 67.3 | 70 |
| fat | 4.3 | 10 |
| Kcal/gm | 3.85 | |

| **Table S3 Identification and quantification of major compounds in CPPP by non-targeted metabolomic analysis** | | | | | |
| --- | --- | --- | --- | --- | --- |
| **No.** | **Compound name** | **Molecular formula** | **Category** | **Molecular weight (Da)** | **Relative content (%)** |
| 1 | γ-Linolenic acid | C₁₈H₃₂O₂ | Fatty acids and derivatives | 296.24 | 11.65 |
| 2 | Azelaic acid | C₉H₁₆O₄ |  | 188.11 | 9.3 |
| 3 | Linoleic acid | C₁₈H₃₂O₂ |  | 280.24 | 1.18 |
| 4 | Isolariciresinol | C₁₈H₂₀O₆ | Lignans | 362.17 | 5.45 |
| 5 | Pinoresinol-glucoside | C₂₂H₂₈O₁₀ |  | 520.2 | 0.68 |
| 6 | D-Fructose | C₆H₁₂O₆ | Saccharides | 180.06 | 3.88 |
| 7 | Sucrose | C₁₂H₂₂O₁₁ |  | 342.12 | 3.17 |
| 8 | Glucose | C₆H₁₂O₆ |  | 180.06 | 2.3 |
| 9 | Sorbose | C₆H₁₂O₆ |  | 180.06 | 2.05 |
| 10 | D-Galactose | C₆H₁₂O₆ |  | 180.06 | 0.34 |
| 11 | Sorbitol | C₆H₁₄O₆ |  | 182.08 | 0.27 |
| 12 | Nystose | C₂₄H₄₂O₂₁ |  | 666.23 | 0.04 |
| 13 | Uridine | C₉H₁₂N₂O₆ | Nucleotides and derivatives | 244.07 | 2.26 |
| 14 | Adenosine | C₁₀H₁₃N₅O₄ |  | 267.1 | 0.93 |
| 15 | Adenine | C₅H₅N₅ |  | 135.05 | 0.87 |
| 16 | Guanosine | C₁₀H₁₃N₅O₅ |  | 283.09 | 0.04 |
| 17 | Luteolin | C₁₅H₁₀O₆ | Flavonoids | 286.05 | 0.98 |
| 18 | Cynaroside | C₂₁H₂₀O₁₁ |  | 448.1 | 0.2 |
| 19 | Diosmetin | C₁₆H₁₂O₆ |  | 300.06 | 0.04 |
| 20 | Lobetyolin | C_20_H_28_O_8_ | Phenolic acids and derivatives | 396.43 | 1.71 |
| 21 | Effuso | C₁₆H₂₀O₂ |  | 251.15 | 1.91 |
| 22 | p-Coumaric acid | C₉H₈O₃ |  | 164.16 | 1.82 |
| 23 | Protocatechuic acid | C₇H₆O₄ |  | 154.12 | 1.66 |
| 24 | Cinnamic acid | C₉H₈O₂ |  | 148.16 | 1.62 |
| 25 | Caffeic acid | C₉H₈O₄ |  | 180.16 | 1.6 |
| 26 | Ferulic Acid | C₁₀H₁₀O₄ |  | 194.18 | 1.56 |
| 27 | Cryptochlorogenic acid | C₁₆H₁₈O₉ |  | 354.31 | 1.54 |
| 28 | Neochlorogenic acid | C₁₆H₁₈O₉ |  | 354.31 | 1.36 |
| 29 | Tangshenoside I | C₂₉H₄₂O₁₈ |  | 678.64 | 1.33 |
| 30 | Chlorogenic acid | C₁₆H₁₈O₉ |  | 354.31 | 1.31 |
| 31 | Syringin | C₁₇H₂₄O₉ |  | 372.36 | 1.25 |
| 32 | 4-Hydroxybenzaldehyde | C₇H₆O₂ |  | 122.12 | 0.59 |
| 33 | Feruloylquinic acid | C₁₇H₂₀O₉ |  | 368.34 | 0.43 |
| 34 | Catechol | C₆H₆O₂ |  | 110.11 | 0.35 |
| 35 | 3-O-Feruloylquinic acid | C₁₇H₂₀O₉ |  | 368.34 | 0.32 |
| 36 | Protocatechuic Aldehyde | C₇H₆O₃ |  | 138.12 | 0.26 |
| 37 | 6,7-Dihydroxycoumarin | C₉H₆O₄ |  | 178.14 | 0.22 |
| 38 | Quinic acid | C₇H₁₂O₆ | Organic acids | 192.17 | 1.71 |
| 39 | Succinic acid | C₄H₆O₄ |  | 118.03 | 0.47 |
| 40 | Malic acid | C₄H₆O₅ |  | 134.02 | 0.07 |
| 41 | Atractylenolide Ⅲ | C_15_H_20_O_3_ | Terpenoids | 248.32 | 1.26 |
| 42 | Oleanolic acid | C₃₀H₄₈O₃ |  | 456.26 | 0.42 |

**Figure S1. Alpha diversity of gut microbiota across all groups. (A) Chao1 index. (B) Shannon index.**
